# Calibration of Stereo Pairs Using Speckle Metrology

**DOI:** 10.3390/s22051784

**Published:** 2022-02-24

**Authors:** Éric Samson, Denis Laurendeau, Marc Parizeau

**Affiliations:** Electrical and Computer Engineering, Faculty of Science and Engineering, Université Laval, Quebec City, QC G1V 0A6, Canada; eric.samson.2@ulaval.ca (É.S.); marc.parizeau@gel.ulaval.ca (M.P.)

**Keywords:** camera calibration, stereo reconstruction, laser speckle, speckle metrology

## Abstract

The accuracy of 3D reconstruction for metrology applications using active stereo pairs depends on the quality of the calibration of the system. Active stereo pairs are generally composed of cameras mounted on tilt/pan mechanisms separated by a constant or variable baseline. This paper presents a calibration approach based on speckle metrology that allows the separation of translation and rotation in the estimation of extrinsic parameters. To achieve speckle-based calibration, a device called an Almost Punctual Speckle Source (APSS) is introduced. Using the APSS, a thorough method for the calibration of extrinsic parameters of stereo pairs is described. Experimental results obtained with a stereo system called the Agile Stereo Pair (ASP) demonstrate that speckle-based calibration achieves better reconstruction performance than methods using standard calibration procedures. Although the experiments were performed with a specific stereo pair, such as the ASP, which is described in the paper, the speckle-based calibration approach using the APSS can be transposed to other stereo setups.

## 1. Introduction

The objective of stereovision in the context of *metrology* is to reconstruct the accurate 3D coordinates of a point *P* in space using right and left images *P_l_* and *P_r_* of *P* when observed by cameras situated at different locations. Stereo reconstruction requires that (*i*) a model of the stereo system is available and (*ii*) the parameters of the model can be estimated with accuracy. The model of a *stereo system* is comprised of two components: (*i*) a *camera* model (described by the intrinsic parameters) and (*ii*) the rigid transformation giving the position and orientation of one camera in the reference frame of the other (the extrinsic parameters).

When both cameras are immobile during image acquisition, they form a *static* stereo pair; however, stereovision is not limited to static systems and 3D stereo reconstruction is possible when the cameras form an *active* stereo pair. The concept of active vision, which was introduced in 1988 by Aloimonos [1] and Bajcsy [2], consists of exploring an environment by moving the sensors.

Three requirements must be met by active stereo systems to achieve accurate 3D reconstruction. Firstly, the mechanism responsible for moving the cameras must achieve high accuracy and repeatability. Secondly, the model describing the stereo system must be as faithful as possible to the real “physical” system. Thirdly, a calibration technique must be available to estimate the parameters of the model.

This paper presents a detailed procedure for the calibration of extrinsic parameters of stereo pairs for 3D reconstruction in metrology applications. While the focus is on active stereo pairs (i.e., systems for which the cameras are mounted on tilt/pan mechanisms and potentially on translation stages for dynamic baseline adjustment), the approach also extends to static pairs. A pinhole model is generally used for the cameras mounted in tilt/pan mechanisms. When the tilt/pan mechanism is rotated to steer the optical axis of the camera, the camera also undergoes a translation since it is impossible to align the center of projection with the center of rotation of the mechanism. This translation is generally left uncalibrated. Yet, even if this translation is small, it can impact the accuracy of 3D reconstruction significantly.

A calibration method based on speckle metrology is presented to uncouple rotation from translation and allows for accurate calibration of extrinsic parameters of active stereo pairs. The method relies on a device called Almost Punctual Speckle Source (APSS). To illustrate the efficiency of the proposed speckle-based calibration approach, experiments were conducted on an active stereo pair called Agile Stereo Pair (ASP); however, the approach can be applied to other designs exploiting cameras mounted on tilt/pan mechanisms.

The paper is structured as follows. A literature review on active stereo pairs and multi-camera systems in the context of metrology is presented in Section 2. The review demonstrates that accurate calibration is not a widespread practice and that there is a need for a systematic approach for calibration that considers all parameters carefully.

Then, the stereo pair used for the experiments is presented in Section 3. This device, called the “Agile Stereo Pair” (ASP), shares common features (tilt/pan mechanisms for steering the cameras, adjustable baseline) with many active stereo systems. The purpose of presenting the ASP before the calibration procedure is to cover the different intrinsic and extrinsic parameters that need to be calibrated accurately. Although the calibration procedure can be extended to other stereo pairs, one must be used to demonstrate the ability of the calibration procedure to reveal details that need to be taken into account in the calibration.

The procedure for the calibration of the intrinsic/extrinsic parameters of active stereo pairs follows in Section 4. Although the approach shares common steps with other approaches published in the literature, it proposes a four-step procedure for the calibration of the transform between the camera reference frame and the reference frame attached to the tilt/pan mechanism. This transform, which is key to accurate 3D reconstruction, is ignored by existing techniques. The four-step procedure exploits speckle metrology to separate translation from rotation and to calibrate extrinsic parameters associated with this transform. The basic principles of speckle metrology are introduced, and a calibration device called the Almost Punctual Speckle Source (APSS) is described and exploited by the procedure. 

In Section 5, results obtained by an exhaustive set of experiments demonstrate the efficiency of the speckle-based calibration method and the impact of calibration on 3D reconstruction.

The paper concludes with an overview of the proposed method and proposes future work.

## 2. Overview of Active Stereo Pairs and Calibration Approaches

In this section, an overview of active stereo pairs and multi-camera systems for 3D reconstruction and tracking is presented. The focus is placed on the calibration of such systems since accurate calibration is key to the use of vision systems for metrology applications.

One of the first active stereo pairs was proposed by Krotkov et al. [3] to emulate the human visual system. This resulted in a system with 11 degrees of freedom (DOF). Unfortunately, the stereo pair achieved poor accuracy and repeatability due to the use of belts and gearboxes for camera steering and potentiometers for measuring angular motion.

The KTH Robotics Head (Pahlavan et al. [4]) was a step forward in the design of biologically inspired stereo systems. The KTH Head implements 13 DOFs: independent rotation for each camera, baseline adjustment, tilt and pan of a “neck”, motorized lenses for zooming, focus adjustment and aperture control, and alignment along the optical axis of each camera through a linear stage (2 DOFs). A calibration procedure is described, but the paper does not provide results on its accuracy.

Urquhart et al. have proposed a stereo pair for which the main design principle was to achieve accurate 3D reconstruction [5]. Specifications for the system were formulated in terms of maximal 3D reconstruction error inside the workspace of the stereo pair. This work demonstrated clearly that the mechanical system must be assembled with accuracy. As a result, the number of degrees of freedom must be kept to a minimum. Urquhart’s design implements 2 DOFs in rotation per camera; however, the paper does not report any results on 3D reconstruction accuracy, and no calibration procedure is described.

It appears that the development of active stereo pairs has slowed down between 1988 and 1992. Reviews of some systems not mentioned above may be found in Milios et al. [6] and Wavering et al. [7].

In contrast with the 1988–1992 period, the year 1993 was very prolific since at least five new active vision designs were presented in the literature. These systems are described in detail in Sharkey et al. [8,9], Milios et al. [6] for the TRISH stereo system, Wavering et al. [7] for the TRICLOPS system, and Ferrier et al. [10] and Crowley et al. [11] for the LIFIA system. For all these systems, the research has focused on increasing the dynamic performance, as well as flexibility and compactness. System accuracy issues were also of concern but, except for [6], were not addressed as formally as in Urquhart et al. [5].

After this period, new active stereo systems were introduced, but at a much slower pace. ESCHeR [12], HyDrA [13], CeDAR [14,15,16], and the BMC robotic head [17] are instances of such systems. As mentioned further in this section, several *multi-camera* systems not necessarily dedicated to 3D reconstruction have also been proposed.

The review of active *stereo* systems reveals that although major efforts were devoted to the design of very accurate/high precision systems, very few papers report results on measurement accuracy because 3D reconstruction with an active stereo pair requires that the extrinsic parameters be known with accuracy and, consequently, be calibrated. Among the stereo pairs described above, only four rely on a calibration procedure of some sort. ESCHeR focuses on calibrating the lenses and the vergence angle between the cameras [18]. There is no evidence of baseline calibration and errors in mechanical alignment of the systems seem to be neglected.

Sophisticated calibration procedures are proposed for the KTH and LIFIA pairs. The approach for calibrating the KTH pair relies on a detailed model of the mechanical system. Instead of using a calibration target, the approach for calibrating the LIFIA pair aims at providing updates for the extrinsic parameters while the system is operating. The updates are computed from the observation of specific stationary features in the scene.

IIS is an active stereo system for which a calibration procedure is described [19]. The IIS pair, which includes a motorized lens, has been designed specifically for the purpose of 3D reconstruction and a sophisticated calibration technique has been implemented. It is based on a detailed model of the mechanical structure of the system and allows for the calibration of the 35 kinematic parameters of the stereo head. The calibration uses a nine-disc planar target for which the 3D coordinates of the centroids have been measured with a Coordinate Measuring Machine (CMM). This departs from the approach presented in this paper, which uses a 3D (i.e., not planar) target.

In addition to the above, several other approaches have been proposed for the calibration of multi-camera vision systems not entirely targeting stereo reconstruction. It is well known that the most straightforward method to calibrate cameras is by means of a target with known coordinates.

Traditionally, a planar pattern or a one-dimensional stick with distinct markers and constant position in a reference frame is employed and is considered as a passive target, which may cause inaccuracy in the localization of its feature points by image processing algorithms. An active target results in more flexibility of the calibration process and refers to a pattern implemented on a digital display or moving an LED as a marker [20,21].

As Kurillo et al. [22] proposed, both active and passive target types can be used jointly for calibration. It is a method for a multi-camera setup of distributed cameras with the constraint that the two cameras must share a common field of view. All intrinsic parameters are estimated using Tsai’s [23] method. Epipolar geometry and bundle adjustment are used to calibrate the extrinsic parameters. The approach was tested with real data on twelve Dragonfly cameras. A checkerboard and two markers placed at each end of a metal bar are used as the calibration target. The proposed approach does not require prior information regarding the positions of cameras.

In addition to the papers discussed above, another approach for calibrating a multi-camera network, using both target types, was presented by Beriault et al. [24]. In the first step, the initial values of the intrinsic parameters and lens distortion are estimated for each camera separately with Tsai’s [23] and Zhang’s [25] algorithms. As the authors explain, a reliable estimation of the intrinsic parameters requires twenty images of the checkerboard target from different viewpoints. A stick with a Light-Emitting Device (LED) mounted at its end is waved in space arbitrarily as a target for the calibration of the extrinsic parameters. Considering its unknown 3D coordinate, image correspondences between pairs of cameras are exploited for pairwise feature point matching and fundamental matrix calculation. Based on Hartley and Zisserman [26], decomposition is performed on each fundamental matrix to result in a rotation matrix and translation vector. All estimated extrinsic parameters are unified with a weighted graph of cameras to find a rotation and translation binding all the cameras in the stereo vision network. Afterwards, with the help of sparse bundle adjustment, the estimated values are refined to reach a more precise calibration. The proposed method was validated on a system composed of eight Flea2 1394b cameras. A reprojection accuracy of ½ pixel was reported.

A combination of photogrammetric and self-calibration methods for a multi-camera system is proposed by Loaiza et al. [27]. The former method is used to estimate the intrinsic parameters of each camera individually, and the latter helps in the estimation of the extrinsic parameters. A one-dimensional pattern called “projective invariant pattern” enables the stereo vision system to acquire data on more than one pattern and its collinear markers simultaneously in real-time. By applying Zhang’s method [25], intrinsic parameters and lens distortion coefficients are estimated first. Furthermore, the self-calibration step for finding the initial value of extrinsic parameters consists of extracting the essential matrix from the camera fundamental matrix [28]. Taking collinearity of the markers into account, parameters are then optimized by defining control functions to ensure the correctness of the estimated value. An average reconstruction error of a projective invariant pattern (set of active light markers mounted on a linear wooden stick, pattern 1: AB = 39.01 mm, BC = 140.196 mm, CD = 99.156 mm; pattern 2: AB = 99.65 mm, BC = 139.505 mm, CD = 180.565 mm) of 1.5 mm is reported based on experiments conducted on two different sets of cameras. The first set is composed of four firewire cameras and the second includes two firewire and two Logitech webcams.

A one-dimensional target can improve efficiency due to its simple shape. Sun et al. [29] presented a calibration technique based on a 1D target with an unknown motion for a stereo pair consisting of two pinhole cameras. By extracting some feature points placed on the two targets with known intervals and finding their vanishing point in the images captured from different angles, intrinsic and extrinsic parameters are estimated. All parameters are then optimized by minimizing the error between the real and image coordinates of the feature points. Moreover, rotation and translation parameters are estimated using another 1D target with only two feature points with known distance from each other. Lastly, global optimization of all parameters is achieved by the Levenberg–Marquardt algorithm. The paper reports global optimization as an improvement on the error measured between the real distance and the estimated distance between feature points (located at about 3500 mm from the stereo pair and spaced by 1218.64 mm) from 0.154 to 0.046 mm, as tested on a stereo pair comprised of two Canon EOS-5D cameras. Furthermore, the target can be projected on the field of view of the cameras in the stereo vision system. The absence of a physical target makes the calibration procedure suitable for industrial or on-site applications.

The approach proposed by Zhao et al. [30] utilizes a cross-shaped phase-shifting sinusoidal pattern projected onto a surface as the target for the calibration of extrinsic parameters, while intrinsic parameters, determined by Zhang’s method [25], are considered to be known and stable during the whole process. To achieve better accuracy, many corresponding pairs of points are retrieved by means of matches with the heterodyne multi-frequency phase-shifting method. Bundle adjustment is then used to optimize the extrinsic parameters. The method was evaluated on a system consisting of two Basler acA2500-14 gm CMOS cameras and is reported to have less than 3 s processing time and RMS error of 0.025 mm in a measurement region of 1200 mm × 1000 mm.

Spheres can act as calibration targets as they are rotationally symmetric. Shen and Hornsey [31] exploited spheres as targets for a multi-camera network consisting of twelve inward-looking cameras. To determine the accurate position of each sphere, the target is scanned with a 3D laser scanning system. The method to estimate the intrinsic parameters is based on the approach presented by Zhang et al. [32]. This method takes accounts for the relationship between the dual images of the target spheres and the dual image of the absolute conic and Challis’s method [33] for computing the extrinsic parameters. As a proof of concept, the method proposed in [31] was applied to a set of twelve Aptina Imaging MT9V111 ¼ Inch VGA cameras and an average reprojection error of 4.82 pixels was reported for extrinsic parameters. This is an improvement of approximately 3.5 times compared to the previous results reported by Shen et al. [34] with the same calibration technique without accurately measuring the target position with a 3D scanner.

Self-calibration methods exploiting epipolar geometry and images of the scene have been proposed. For instance, Guan et al. use a minimum number of nine images and a RANSAC-based approach to estimate the intrinsic and extrinsic parameters of a stereo pair composed of cameras with radial distortion [35].

The recent advances in machine learning, especially deep learning, have boosted the development of applications using stereo systems for 3D reconstruction. Based on the survey presented in [36], it appears that the cameras of the stereo pairs are calibrated with classical methods described above before being used for 3D reconstruction using deep-based stereo matching. A survey in [37] is a testimony of the increase in popularity of deep learning in stereo reconstruction since it reviews more than 150 papers; however, the issue of camera calibration is not addressed in the survey.

Some authors have proposed deep learning-based approaches for single-camera calibration [38], but the focus is on the definition of a *perceptual* measure of camera calibration for 3D object insertion in virtual scenes, calibration-based image retrieval, and compositing rather than for metrology applications.

The above review shows that active stereovision systems adopt different architectures and that some use complex calibration procedures. The following sections present a detailed procedure for the calibration of active stereo pairs, which can be extended to static pairs. A specific stereo pair is used to demonstrate the efficiency of the calibration approach, which is based on speckle metrology.

## 3. The Agile Stereo Pair (ASP)

This section presents a brief overview of the Agile Stereo Pair (ASP) that is used for the experiments on speckle-based calibration. The ASP includes the same parameters as most stereoscopic pairs; therefore, the calibration method described in Section 4 can be adapted to other mechanisms.

### 3.1. Description of the ASP

**Figure 1:**
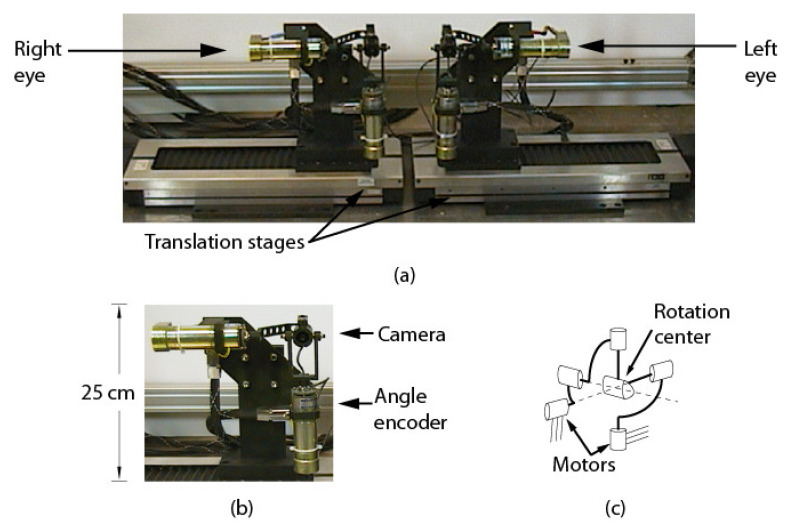
The ASP with both eyes and translation stages (**a**). (**b**) Actual mechanism for the right eye of the camera. The mechanism is very compact. (**c**) Basic principle of the 2DOFs parallel mechanism for tilt/pan rotation.

The ASP is a 6-DOFs system (see Figure 1a) composed of two 2-DOFs parallel mechanisms for tilt/pan rotation (Figure 1b), each mounted on a translation stage for baseline adjustment. The parallel mechanism is the key component of the ASP. Its structure allows the motors to be solidly attached to the base, which makes the system compact, rigid, and repeatable. The mechanical design ensures that the rotation axes cross at a point located at the center of the mechanism (called the end effector) and guarantees, at least theoretically, pure rotation around this point (Figure 1c). As reported in Section 5, the physical implementation of the mechanism does not ensure perfect rotation, a condition that has an impact on calibration.

As demonstrated in Section 4, the projection center of the camera and the center of rotation of the parallel mechanism are not “material” points. It is thus impossible to adjust the position of the camera in the end effector of the mechanism so that they are perfectly aligned. As a result, the parallel mechanism cannot impose pure rotation on *the camera*. This implies that the position and orientation of the camera in the mechanism need to be calibrated accurately since a small calibration error may lead to large errors on stereo reconstruction. Details on the components of the ASP are given in Table 1.

As shown in Table 2, very high dynamic performance with small-size/low-power motors can be achieved by the system because of the small inertia of the mechanism. As reported in [39], the ASP only needs 0.130 s to perform a full-range (80°) saccade movement on its slower axis (tilt) and 0.087 s on its fastest axis (pan). The tilt axis is slower because it must fight gravity. In practical applications, most saccades cover shorter angular intervals since they generally aim at moving the center of attention to a feature that already lies in the field of view of the sensor. Assuming a four-frame interval (i.e., ~0.133 s) between saccades (see Brooks et al. [40]) is enough for the vision system to analyze the new situation before initiating a new saccade and considering the longest possible saccade movement (0.130 s), the ASP can perform at least 3.8 saccades/s. This matches and even outperforms the human eye, which can achieve 3–4 saccades/s [41].

### 3.2. Theoretical 3D Reconstruction Accuracy of the ASP

The accuracy of the 3D reconstruction achieved by the ASP (and similar systems) depends on many factors. Firstly, parameters such as baseline, operating distance with respect to scene objects, focal length, resolution, and pixel size of the cameras must be considered. The accuracy of the approach for calibrating these parameters is also very important. Secondly, the factors specific to the ASP, such as the tilt and pan angles, the resolution of the rotation encoders, the precision of the linear translation stages, and the accuracy of the mechanical system assembly must also be accounted for. Again, the calibration procedure for estimating these additional parameters is of paramount importance.

**Figure 2:**
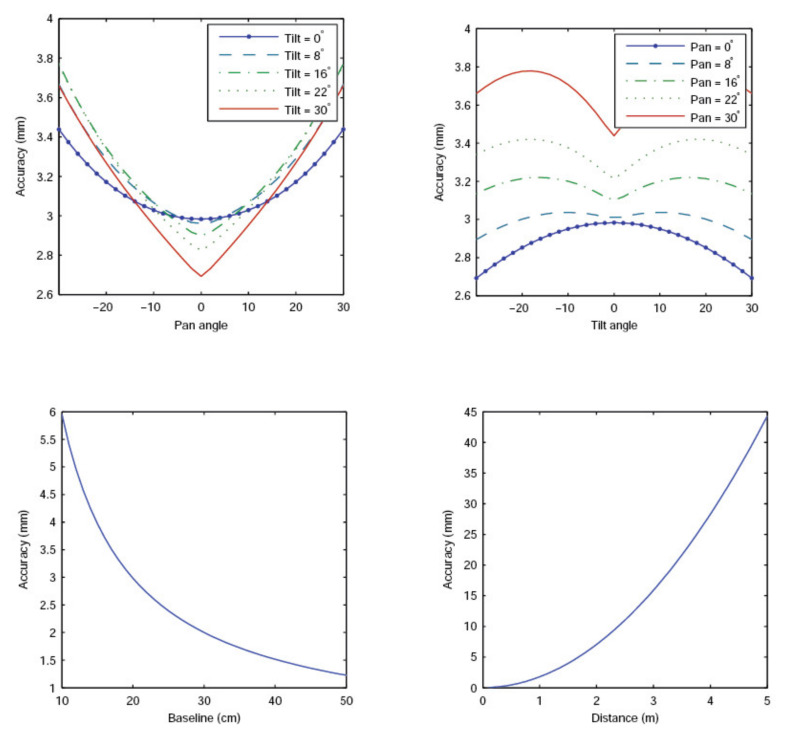
Theoretical reconstruction accuracy with respect to three dynamic parameters (pan, tilt, baseline) and distance. When variables are not given, default values are used: pan = 0°, tilt = 0°, precision of encoders ±0.0055°, baseline = 20 cm, operating distance = 1.5 m, focal length of the cameras = 8 mm, pixel size = 5.7 mm, image resolution = 640 × 480, precision of stereo matching = ±0.1 pixel. Top-left/right: reconstruction accuracy as a function of pan/tilt angle. Bottom left/right: reconstruction error as a function of baseline/distance. Baseline is in cm while Distance to object is in m.

The plots in Figure 2 show the maximum achievable depth reconstruction accuracy of the ASP with respect to the dynamic parameters (tilt, pan, and baseline) and the operating distance when assuming perfect calibration and perfectly rigid mechanical assembly.

The values that were chosen for some of these parameters are pessimistic. For instance, the precision of stereo matching can reach much better results, especially for calibration targets that are designed such as to maximize detection accuracy of the features (often corners of squares) used for matching.

## 4. Calibration of Stereo Pairs and the ASP

As mentioned in Section 1, accurate 3D reconstruction with static/active stereo pairs depends on three factors:

1. *Accuracy* and *repeatability* of the mechanism for active pairs.
2. Quality of the *models* describing the mechanism and cameras.
3. Quality of the *calibration* of model parameters.

The first constraint is satisfied implicitly by the ASP since it exploits a parallel mechanism based on closed kinematic chains. The mechanism being constrained at two points for each kinematic chain, it is very rigid, its motion is highly repeatable and, except for small machining errors, cannot deviate from allowed configurations (e.g., combinations of tilt and pan angles) inside its workspace [42].

The model describing the ASP must be faithful to the actual mechanical and optical design but be simple enough to allow manageable calibration. The geometric model of the cameras and the mechanical model of the ASP are presented in Section 4.1.

The calibration procedure for estimating the parameters of the model is presented in detail in Section 4.2. The limitations of classical calibration techniques for estimating some parameters are discussed and a solution exploiting speckle metrology is presented in Section 4.3.

### 4.1. Geometric Models of the Cameras and the ASP

A standard pinhole camera model and a radial distortion model were adopted for describing the cameras of the ASP. In the following, a lowercase letter refers to a column vector, an uppercase refers to a matrix. A letter headed with symbol “~” refers to a vector/matrix in homogeneous coordinates.

**Figure 3:**
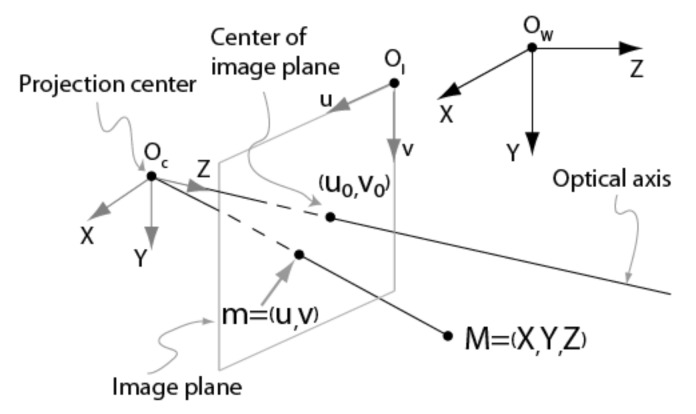
Geometric image formation process. A 3D point *M* in the world reference frame $O_{W}$ is projected on the image plane as point *m* with coordinates (*u*,*v*) in the image reference frame $O_{i}$. The center of the image plane (i.e., the location of the intersection between the optical axis and the image plane) is located at (*u*_0_,*v*_0_) in the reference frame of the image $O_{i}$. The center of projection of the pinhole is at the origin $O_{C}$ of the reference frame attached to the camera.

The geometric image formation process for the pinhole shown in Figure 3, is expressed by Equations (1)–(4).

(1) $$s\tilde{m}=K\left[\begin{array}{cc} Q & t \end{array}\right]{\tilde{M}}_{W}$$

(2) $$K=\left[\begin{array}{ccc} \alpha & \gamma & u_{0}\\ 0 & \beta & v_{0}\\ 0 & 0 & 1 \end{array}\right]$$

(3) $$Q=\left[\begin{array}{ccc} r_{11} & r_{12} & r_{13}\\ r_{21} & r_{22} & r_{23}\\ r_{31} & r_{32} & r_{33} \end{array}\right]$$

(4) $$t={\left[\begin{array}{ccc} t_{x} & t_{y} & t_{z} \end{array}\right]}^{t}$$

where ${\tilde{M}}_{W}={\left[\begin{array}{cccc} X_{W} & Y_{W} & Z_{W} & 1 \end{array}\right]}^{t}$ is a point in 3D, $\tilde{m}={\left[\begin{array}{ccc} u & v & 1 \end{array}\right]}^{t}$ is its projection on the image plane and *s* is a scale factor. *K* is the matrix of intrinsic parameters, *Q* is the rotation matrix and *t* is the translation vector.

The definition of the parameters in Equations (1) and (2) is given in Table 3.

Equations (5) and (6) are combined with the linear model of Equation (1) to account for radial distortion:

(5) $$\overset{˘}{x}=x+x\left[k_{1}\left(x^{2}+y^{2}\right)+k_{2}{\left(x^{2}+y^{2}\right)}^{2}\right]$$

(6) $$\overset{˘}{y}=y+y\left[k_{1}\left(x^{2}+y^{2}\right)+k_{2}{\left(x^{2}+y^{2}\right)}^{2}\right]$$

where $\left(x,y\right)$ and $\left(\overset{˘}{x},\overset{˘}{y}\right)$ are the ideal (without distortion) and actual coordinates of the image point in the normalized image plane, i.e.:

(7) $$\left[\begin{array}{c} x\\ y\\ 1 \end{array}\right]=K^{-1}\left[\begin{array}{c} u\\ v\\ 1 \end{array}\right]\;and\;\left[\begin{array}{c} \overset{˘}{x}\\ \overset{˘}{y}\\ 1 \end{array}\right]=K^{-1}\left[\begin{array}{c} \overset{˘}{u}\\ \overset{˘}{v}\\ 1 \end{array}\right]$$

Tangential distortion is neglected but could be calibrated with the method in [43].

**Figure 4:**
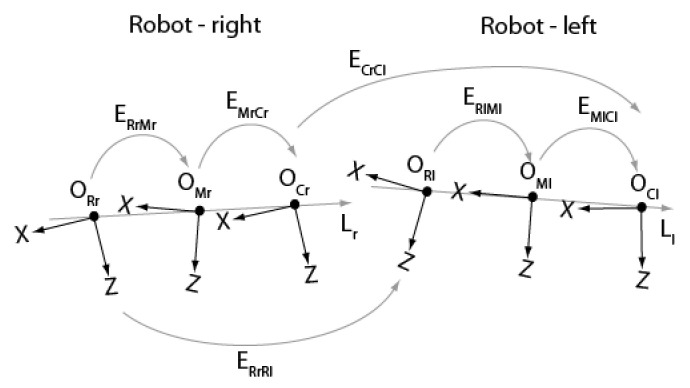
The geometric model of the ASP showing the reference frames describing the stereo setup as well as the transformations between frames. The *Z*-axis of all frames points towards the page. The linear translation stages are represented by Lines $L_{r}$ (**right**) and $L_{l}$ (**left**).

The geometric model of the ASP shown in Figure 4 is comprised of six reference frames and five independent frame transformations. Frame $O_{Ri}$ (*i* = “*r*” for right and “*l*” for left) is the fixed base reference frame for *robot k* (*k* = 1,2). The origin of this frame is located at the point where the axes (X-tilt and Y-pan) of the motors of a parallel mechanism intersect. Axis Z points toward the scene. A *robot* is composed of a *parallel mechanism* and a *translation stage.*

Frame $O_{Mi}$ is attached to the parallel mechanism supporting the camera. This frame can move along the linear stage with orientation $L_{i}$ and rotate around axes X and Y. Before the ASP starts to move, $O_{Mi}$ is perfectly aligned with $O_{Ri}$. Finally, frame $O_{Ci}$ is the reference frame of the camera mounted in the parallel mechanism. It corresponds to frame $O_{C}$ in Figure 3. To compute 3D coordinates, transform $E_{Cr,Cl}$, which defines the pose of the left camera reference frame with respect to the right camera reference frame, must be estimated by calibration. According to the geometric model shown in Figure 4, this transform can be computed with the following equation:

(8) $$E_{CrCl}=E_{MrCr}^{-1}E_{RrMr}^{-1}E_{RrRl}E_{RlMl}E_{MlCl}$$

Transforms on the right-hand side of Equation (8) are either calibrated or computed according to the current state of the ASP, as explained in the following section.

### 4.2. Procedure for Calibrating the ASP

Overall, the 38 parameters listed in Table 4 need to be calibrated for the ASP. In the following, the procedure for calibrating the intrinsic parameters is explained first. Then, the procedure for calibrating the pose of a camera with respect to the calibration target is presented. This is then used for calibrating the direction vectors $L_{i}$ of the translation axes and transform $R_{RrRl}$.

#### 4.2.1. Calibration of the Intrinsic Parameters of the Cameras

**Figure 5:**
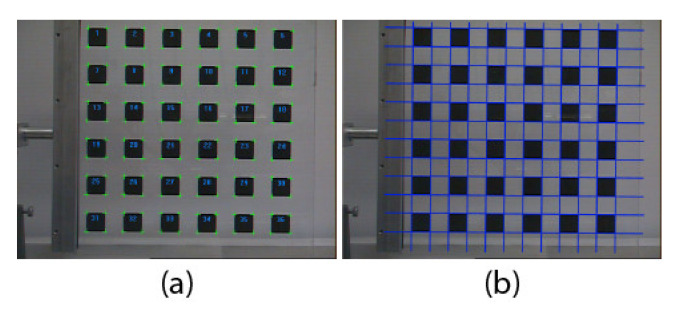
Calibration target composed of a 6 × 6 grid of black squares printed on a transparent glass plate. The side of each square is 2 cm long. The space between the columns and lines in the grid is 2 cm. (**a**) Final result of the detection of the corners of the squares for the first iteration. (**b**) Line fitting for iterations following iteration 1.

The intrinsic parameters of each camera of the ASP are calibrated using Zhang’s method (As a matter of fact, any method for estimating the intrinsic parameters could be used) [24,44] with the calibration target shown in Figure 5. The quality of the calibration depends on the accuracy with which the corners of the squares are detected in the images. To obtain optimal results, Zhang’s method is applied iteratively. At the first iteration, the intrinsic parameters of the cameras are unknown. A sub-pixel detection of the corners of each square in the pattern is performed and a straight-line model is fitted to each side of the square. The intersections between the lines are used as calibration points for Zhang’s algorithm.

Once these first estimates are obtained, the calibration algorithm proceeds iteratively to refine their values. For these iterations, the approach used for detecting the corners of the squares is slightly different. A sub-pixel detection of the sides of each square is again performed. The coordinates of these pixels are corrected for distortion using the set of parameters obtained at the first iteration (and Equations (5) and (6)).

A straight-line model is then fitted to *all* points on the sides of the squares on each line and each column of the grid and the coordinates of the intersection points between these lines are computed (Figure 5b). These refined estimates of the corners are fed to the next iteration of Zhang’s algorithm. For camera lenses with small distortion, this process usually terminates after 4–5 iterations.

#### 4.2.2. Calibration of Transform

For the ASP, transform $E_{RiMi}$, which defines the pose of frame $O_{Mi}$ with respect to $O_{Ri}$, does not need to be calibrated but can rather be *computed* from the current *state* of the ASP. The *state* of the ASP is defined by the following parameters:

1. $\theta_{1}$: current value of the angle of rotation of the motor controlling the longitudinal axis (pan).
2. $\theta_{2}$: current value of the angle of rotation of the motor controlling the latitudinal axis (tilt).
3. λ: the distance that the mechanism has traveled along $L_{i}$.

**Figure 6:**
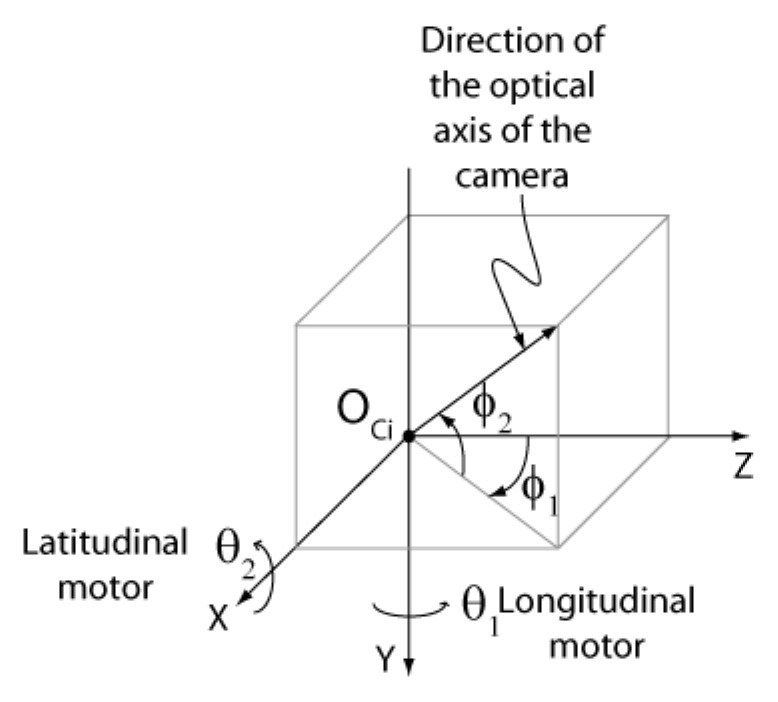
Definition of the angles for the motors and the rotation of the camera reference frame.

The tilt ($\phi_{1}$) and pan ($\phi_{2})$ angles of the camera (frame $O_{Ci}$) are computed with Equations (9) and (10) using the values of the rotation angles of the motors along their respective axis (see also Figure 6 for the definition of $\phi_{1}$ and $\phi_{2}$ [42]:

(9) $$\phi_{1}=\theta_{1}$$

(10) $$\phi_{2}=\tan^{-1}(\tan\left(\theta_{2}\right)/\cos(\theta_{1}))$$

Transform $E_{RiMi}$ can be expressed as compound transform:

(11) $$E_{RiMi}=T_{RiMi}R_{RiMi,y}R_{RiMi,x}$$

where:

(12) $$R{\;}_{RiMi,x}=\left[\begin{array}{cccc} 1 & 0 & 0 & 0\\ 0 & \cos\phi_{2} & -\sin\phi_{2} & 0\\ 0 & \sin\phi_{2} & \cos\phi_{2} & 0\\ 0 & 0 & 0 & 1 \end{array}\right]$$

(13) $$R{\;}_{RiMi,y}=\left[\begin{array}{cccc} \cos\phi_{1} & 0 & \sin\phi_{1} & 0\\ 0 & 1 & 0 & 0\\ -\sin\phi_{1} & 0 & \cos\phi_{1} & 0\\ 0 & 0 & 0 & 1 \end{array}\right]$$

(14) $$T_{RiMi}=\left[\begin{array}{cccc} 1 & 0 & 0 & \alpha l_{xi}\\ 0 & 1 & 0 & \alpha l_{yi}\\ 0 & 0 & 1 & \alpha l_{zi}\\ 0 & 0 & 0 & 1 \end{array}\right]$$

Vector $L_{i}={\left[\begin{array}{ccc} l_{xi} & l_{yi} & l_{zi} \end{array}\right]}^{t}$ in Equation (14) is obtained by calibration. Before describing the procedure for calibrating $L_{i}$, the calibration of the pose of a camera with respect to a calibration target is presented first. This procedure will also be useful for the calibration of matrix $E_{RrRl}$ which expresses the frame transform between the right eye and the left eye.

#### 4.2.3. Calibration of the Pose of a Camera with Respect to a Calibration Target

The estimation of the pose of a camera with respect to a calibration target supposes that the intrinsic parameters have been calibrated using Zhang’s algorithm. Then, assuming that frame $O_{W}$ on the calibration target is chosen so that the points on the corners of the squares all lie on plane Z = 0, Equation (1) can be written:

(15) $$s\tilde{m}=K\left[\begin{array}{ccc} r_{1} & r_{2} & t \end{array}\right]\left[\begin{array}{c} X\\ Y\\ 1 \end{array}\right]$$

where $r_{1}$ and $r_{2}$ are the first two columns of rotation matrix Q. If *H* and $\tilde{M}$ are defined as:

(16) $$H=K\left[\begin{array}{ccc} r_{1} & r_{2} & t \end{array}\right]$$

(17) $$\tilde{M}={\left[\begin{array}{ccc} X & Y & 1 \end{array}\right]}^{t}$$

Equation (15) becomes:

(18) $$s\tilde{m}=H\tilde{M}$$

Homography *H* is a 3 × 3 matrix that is estimated by minimizing the reprojection error:

(19) $$min_{H}=\overset{N}{\underset{k=1}{\sum }}∥m_{k}-{\hat{m}}_{k}{∥}^{2}$$

using a Levenberg–Marquardt non-linear optimization algorithm [25,44]. In Equation (18), $m_{k}={\left[\begin{array}{cc} u_{k} & v_{k} \end{array}\right]}^{t}$ is the image of the *k*th point on the target while ${\hat{m}}_{k}={\left[\begin{array}{cc} {\hat{u}}_{k} & {\hat{v}}_{k} \end{array}\right]}^{t}$ is the image of the projection of the same point using *H*. Once *H* is obtained, rotation matrix *Q* and translation vector *t* are extracted from the matrix using Equations (20)–(24):

(20) $$r_{1}=\lambda K^{-1}h_{1}$$

(21) $$r_{2}=\lambda K^{-1}h_{2}$$

(22) $$r_{3}=r_{1}\times r_{2}$$

(23) $$t=\lambda K^{-1}h_{3}$$

(24) $$\lambda =\frac{1}{∥K^{-1}h_{1}∥}=\frac{1}{∥K^{-1}h_{2}∥}$$

Since $r_{1}$, $r_{2}$ and $r_{3}$ are estimated from real data, matrix $\left[\begin{array}{ccc} r_{1} & r_{2} & r_{3} \end{array}\right]$ does not respect the orthogonality property of rotation matrices. A pure rotation matrix can be obtained through an SVD, as suggested in [44]. Doing so will cause the optimization criterion in Equation (18) to no longer be satisfied. Moreover, the point ${\hat{m}}_{k}$ in Equation (18) is computed without considering the known distortion parameters. To resolve both issues, the set of parameters $\Omega =\left\{\begin{array}{cccccccc} \theta & \psi & \gamma & t_{x} & t_{y} & t_{z} & k_{1} & k_{2} \end{array}\right\}$ is optimized (parameters θ, ψ, and γ define the rotation matrix (Euler angles are used)) using the following new optimization criterion:

(25) $$min_{\Omega }=\overset{N}{\underset{k=1}{\sum }}∥m_{k}-{\hat{m}}_{k}∥{}^{2}$$

where ${\hat{m}}_{k}=Proj\left(\begin{array}{ccccc} K, & k_{1}, & k_{2}, & \Omega , & M_{k} \end{array}\right)$ are the projections of the target points using the camera model, including radial distortion. The results obtained from Equation (20) to Equation (24) are used to provide initial values to the optimization procedure using Equation (25).

#### 4.2.4. Calibration of Translation Vector $L_{i}$

**Figure 7:**
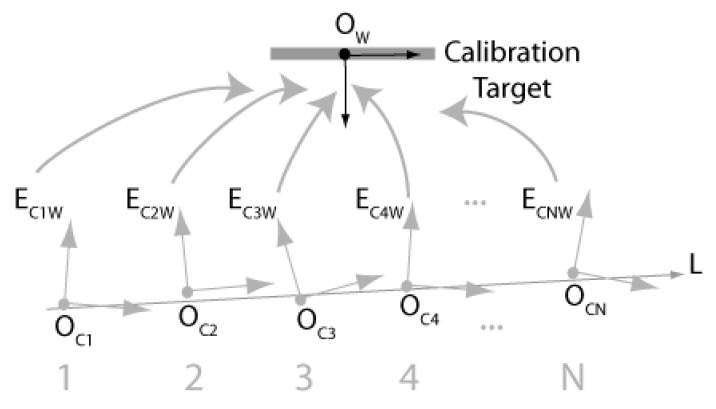
Calibration of translation vector *L*. The pose of the camera reference frame $O_{Ci}$ (for *i* = 1…*N*) with respect to the reference frame of the calibration target $O_{W}$ is estimated with the procedure described in the preceding section. The direction of the line, which is the estimate of L, is obtained from the principal component analysis of the set of positions $O_{C1}$ to $O_{CN}$.

This section describes the procedure for calibrating $L_{i}$, the direction vector of the translation stages of the ASP. Since the procedure is similar for both eyes, indexes *r* and *l* are dropped from the equations. Basically, the procedure consists of estimating the pose of the camera moving along $L_{i}$ with respect to a motionless calibration target. Consequently, it reduces to applying the calibration procedure described in the preceding section to several positions of the camera along the translation stage and then to estimate the direction of $L_{i}$ through a principal components analysis. Figure 7 illustrates the procedure.

#### 4.2.5. Calibration of Transform $E_{RrRl}$

The calibration of $E_{RrRl}$ is equivalent to the well-known problem of calibrating the extrinsic parameters of a standard static stereo pair. Indeed, this transform can be determined by calibrating $E_{CrCl}$ with the ASP being in a general configuration. Knowing $E_{CrCl}$ matrix $E_{RrRl}$ can be found easily by rearranging Equation (8):

(26) $$E_{RrRl}=\begin{array}{ccccc} E_{RrMr} & E_{MrCr} & E_{CrCl} & E_{MlCl}^{-1} & E_{RlMl}^{-1} \end{array}$$

By choosing to calibrate $E_{CrCl}$ with the ASP in its initial configuration, Equation (26) is simplified since, in this case, $E_{RrMr}=E_{RlMl}=I$:

(27) $$E_{RrRl}=\begin{array}{ccc} E_{MrCr} & E_{CrCl} & E_{MlCl} \end{array}$$

**Figure 8:**
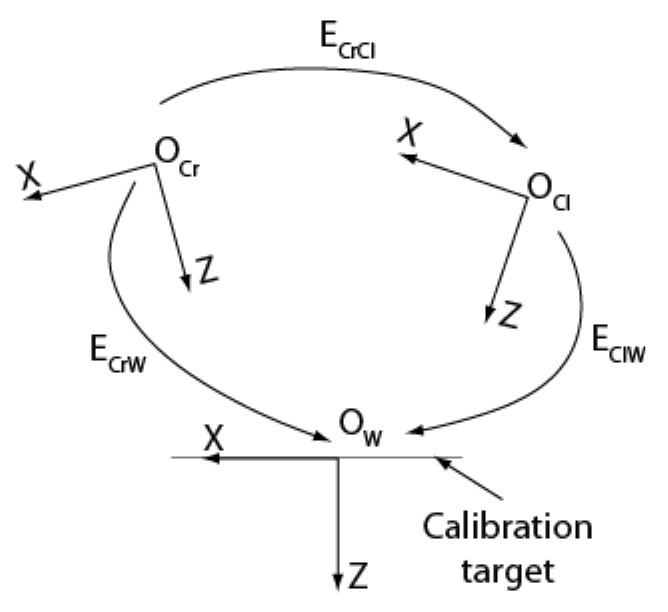
General principle for calibrating a standard stereo pair.

The procedure for calibrating $E_{CrCl}$ is illustrated in Figure 8. Both cameras observe a calibration target and the procedure described above is used to estimate transforms $E_{CrW}$ and $E_{ClW}$ which give the pose of frame $O_{W}$ attached to the calibration target with respect to camera frames $O_{Cr}$ and $O_{Cl}$.

Transform $E_{CrCl}$ can be written as:

(28) $$E_{CrCl}=\begin{array}{cc} E_{CrW} & E_{ClW}^{-1} \end{array}$$

**Figure 9:**
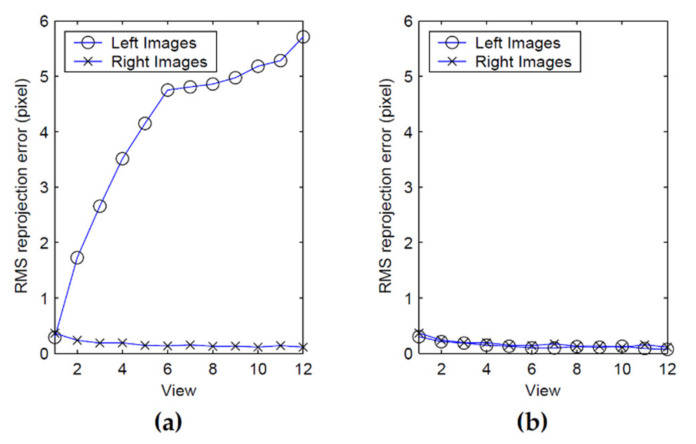
RMS reprojection error before (**a**) and after (**b**) the application of the calibration procedure for *E_C__r__C__l_* as a function of the image number index. This result shows that the reprojection error is small and, more importantly, is the same for the left and right images.

*N* stereo pairs of images of the calibration target are acquired from different positions in the workspace of the ASP. One pair is selected for estimating $E_{CrWi}$ and $E_{ClWi}$ from which an initial value for $E_{CrCl}$ is obtained using Equation (28). Starting with this initial value, the parameters of $E_{CrCl}$ are optimized by minimizing the reprojection error using the remaining pairs. For *N* views with *M* calibration points, the quantity to minimize is:

(29) $$min_{\Omega }\overset{N}{\underset{i=1}{\sum }}\overset{M}{\underset{j=1}{\sum }}∥m_{ijr}-{\hat{m}}_{ijr}∥{}^{2}+∥m_{ijl}-{\hat{m}}_{ijl}∥{}^{2}$$

where Ω is the set of parameters in $E_{CrCl}$ and

(30) $${\hat{m}}_{ijr}=Proj\left(\begin{array}{ccccc} K_{r}, & k_{1r}, & k_{2r}, & E_{CrWi}, & M_{ij} \end{array}\right)$$

(31) $${\hat{m}}_{ijl}=Proj\left(\begin{array}{cccccc} K_{l}, & k_{1l}, & k_{2l}, & E_{CrCl}, & E_{CrWi}, & M_{ij} \end{array}\right)$$

are the projections of target points on the right and left image planes using the estimates for $E_{CrWi}$, $E_{ClWi}$, and $\;E_{CrCl}$. It is worth noting that for the right-hand side camera, all *N* transforms $\;E_{CrWi}$ are used for transforming target points in the camera reference frame. For the left-hand side camera, this frame transformation is achieved through the combination of $\;E_{CrCl}$ with each of the *N* transforms $\;E_{CrWi}$ with Equation (28). Consequently, the reprojection error takes all the views into account in the optimization problem and makes the solution found for $\;E_{CrCl}$ adapted to the entire workspace, as shown in Figure 9. The number of parameters to optimize also increases since the parameters of all *N* transforms $\;E_{CrWi}$ must be considered in Equation (29).

Finally, the geometric model of the ASP is completed by matrix $E_{MiCi}$, which describes the pose of the camera with respect to the parallel mechanism. The parameters of this matrix are also obtained by calibration; however, since frame $O_{Ci}$ is generally very close to frame $O_{Mi}$, accurate calibration of this matrix is very difficult to achieve with classical methods.

### 4.3. Calibration of Transform $E_{MiCi}$ Using Speckle Metrology

**Figure 10:**
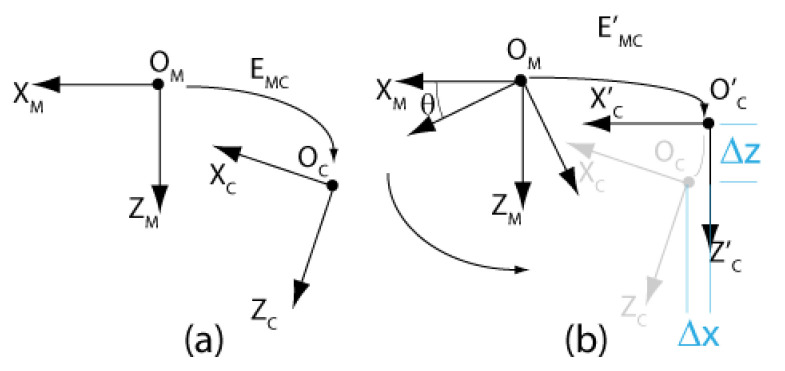
Transform $E_{MC}$ between the manipulator and the center of the camera (**a**). When the manipulator rotates around axis $O_{M}$, the camera rotates and translates by Δx and Δz, which causes errors in the estimation of 3D coordinates from stereo (**b**). The translation is very difficult to estimate using standard calibration techniques.

Figure 10 shows what happens when a manipulator of the ASP rotates around its *Y*-axis with transform $E_{MC}$ not equal to the identity matrix. The rotation causes the camera to rotate by angle θ and to translate by Δx and Δz. As demonstrated in Section 5, the error caused by assuming $E_{MC}=I$ can reach as much as several millimeters at 1 m. Such an error cannot be tolerated if accurate measurements are to be obtained from stereo reconstruction for metrology applications.

Current calibration approaches fail to estimate small transforms because it is difficult to uncouple translation from rotation in the non-linear minimization process. This section describes how speckle metrology techniques can be used to calibrate $E_{MC}$ in the ASP. This calibration approach departs from other techniques both on basic principles and on implementation. Although speckle metrology is used for the calibration of the ASP, it can be extended to other tilt/pan mechanisms and stereo pairs in general.

The ASP is a typical use case that demonstrates the relevance of speckle metrology for camera calibration.

#### 4.3.1. What Is Laser Speckle?

**Figure 11:**
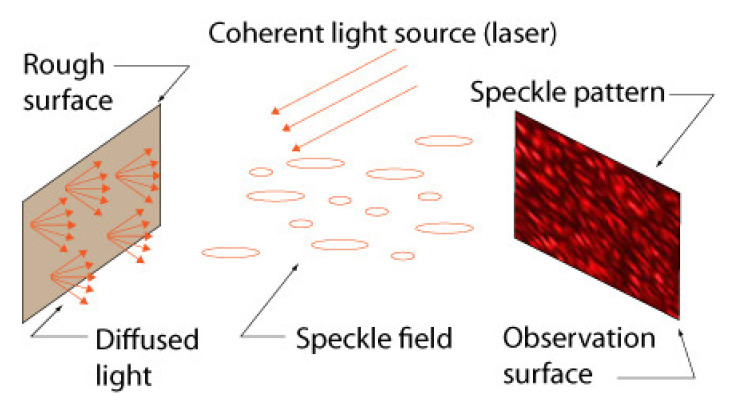
Observation of the objective phenomenon. A coherent light source (laser) illuminates a rough surface that diffuses light in open space leading to the construction of a speckle field. The speckle field can be observed by placing an observation plate in front of the illuminated surface.

Laser speckle is an interference phenomenon that occurs when a source of coherent light illuminates a “rough” surface, the surface roughness being greater than the wavelength of the source. Light waves reflected by this surface interfere and form a non-uniform distribution of intensity in space called a speckle field (see Figure 11). The “3D” distribution of the speckle field is a function of both the source and the surface but remains static (i.e., does not change shape) if the source and the surface do not move relative to each other. Speckle can be observed by placing a *screen* (a piece of white cardboard, for instance) in front of the surface. The speckle field then forms a *speckle pattern* on the surface as shown in Figure 11. The speckle observed in this case is called “objective” speckle.

Another way of observing speckle consists of placing a lens between the surface illuminated by the coherent source and the observation screen, which can be the *image plane* of a camera.

**Figure 12:**
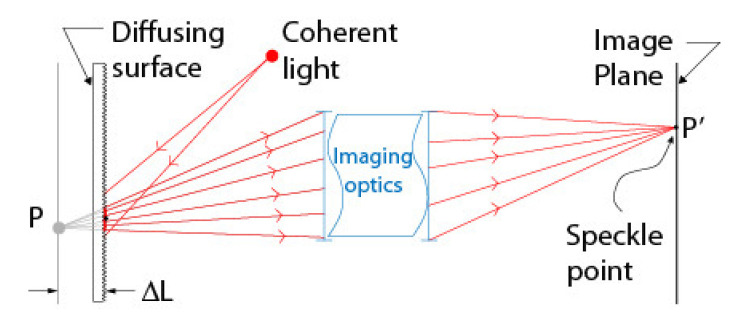
Basic principle of defocused subjective speckle. A diffusing surface is illuminated by a coherent light source. A spot on the surface (corresponding to in-focus point *P*) is imaged on the image plane as point *P′* by the optics of the camera. Speckle point *P′* results from the combination of waves coming from the spot on the diffusing surface. In focus *subjective speckle* is obtained when *D* *L* = 0. In this case, speckle point *P′* results from the combination of waves coming from the single point *P* on the diffusing surface.

In this case, one refers to *in-focus subjective speckle* when the surface, the lens, and the image plane are positioned such that the laws of image formation are respected (i.e., all light rays arriving at a given point on the image plane originate from a single point on the diffusion screen). When it is not the case, one refers to *defocused subjective speckle* (see Figure 12).

A detailed description of speckle properties can be found in [45]. Among these properties, an important one is that a speckle pattern changes as a function of the displacement or deformation of the surface observed either with a screen, for objective speckle, or with a lens (i.e., camera), for subjective speckle. More importantly, depending on the chosen optical configuration, defocused subjective speckle can be made selective to rotations only. This means that defocused subjective speckle can be used for measuring rotations of the observed surface by simply tracking the displacement of points *P* of the pattern (Figure 12) even though the surface undergoes other transformations (e.g., translation) at the same time.

**Figure 13:**
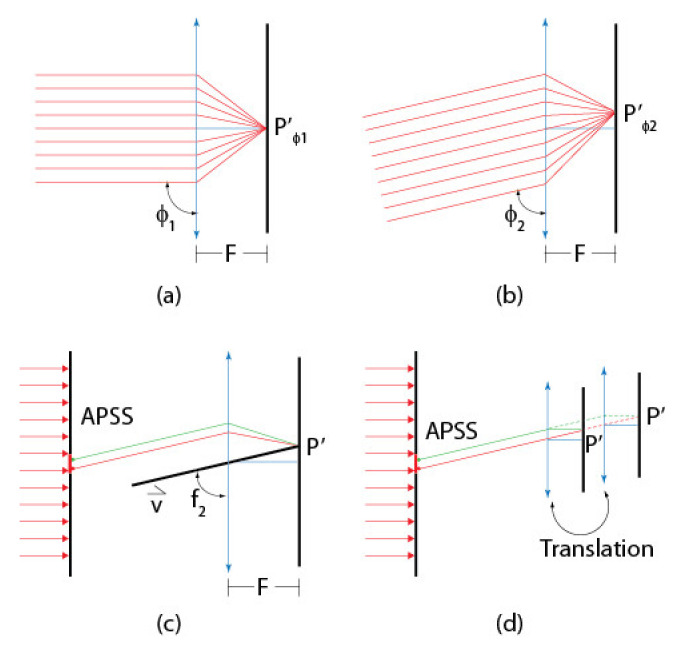
Using defocused speckle for measuring rotations. Collimated light at orientation $\phi_{1}$ generates a point $P\prime_{\phi 1}$ on the speckle pattern (**a**). When the collimated light originates from orientation $\phi_{2}$ relative to the optical axis, the speckle point moves to position $P\prime_{\phi 2}$ on the image plane (**b**). The mapping between orientation and position is encoded by direction vector $\vec{v}$ (**c**). A translation does not affect the orientation-position mapping (**d**). The APSS in the figure is a source of speckle (see text).

This phenomenon has first been demonstrated by Tiziani [46] for the special case of a speckle pattern observed in the focal plane of a convex lens and can be explained by ray optics principles. As illustrated in Figure 13a,b, parallel rays passing through the lens are focused at a single point on its focal plane. The specific position of the point of convergence on the focal plane depends on the angle of incidence of the beam of parallel rays. As the intensity of a particular point of a speckle pattern is the result of the combination of all rays reaching that point, it can be said that a particular point of a speckle pattern originates from the combination of all rays in the speckle field that reach the lens with the same angle of incidence. In other words, the lens transforms *orientation* information in the speckle field into *position* information on the focal plane. This mapping between orientation and position is encoded in the direction vector $\vec{v}$ between a point *P’* on the speckle pattern and the center of the lens (Figure 13c).

Furthermore, Figure 13d shows that a translation of the camera does not affect this orientation–position encoding since the position of convergence only depends on the angle of incidence of parallel rays and not on where they reach the lens. Consequently, exploiting defocused subjective speckle allows uncoupling rotation from translation when calibrating a camera since *rotating* the camera in front of the speckle field causes the speckle pattern to *move* while *translating* the camera leaves the pattern *unchanged*.

The calibration of $E_{MC}$ exploits this important property of defocused subjective speckle. More precisely, and as described next, returning to Figure 10b, the translation component of $E_{MC}$ is estimated once the rotation component is obtained by analyzing the displacement of the speckle pattern on the image plane resulting from the rotation; however, for the method to be effective, both transforms (translation and rotation) must be observed simultaneously.

#### 4.3.2. Basic Principle of Measuring Translation and Rotation Using Speckle

**Figure 14:**
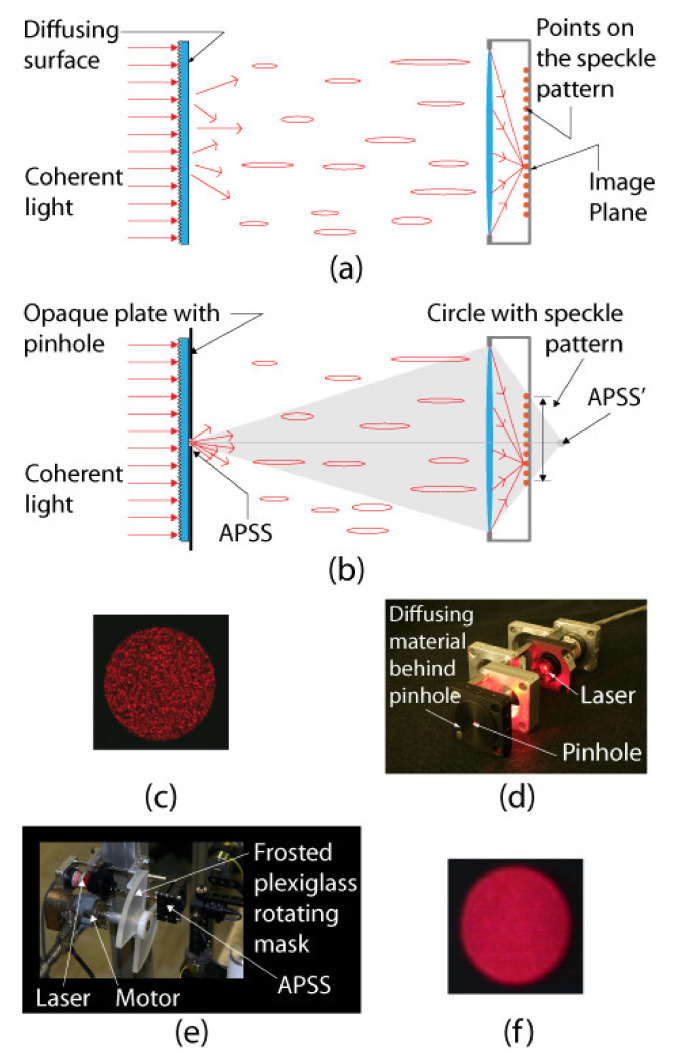
Speckle pattern observed by back illumination of a diffusing surface with a coherent light source (**a**). Placing a mask pierced with a pinhole in front of the diffusing surface creates an Almost Punctual Speckle Source (APSS) observed as a circle filled with speckle on the image plane of the camera (**b**). Front view of the circle in (**b**) with speckle pattern (**c**). The practical implementation of the APSS. The pinhole is used for producing the speckle pattern (**d**) while the rotating mask (**e**) is used for producing the calibration reference point (**f**).

On the one hand, for measuring translation, a camera must observe a set of reference points. These reference points can be markers on a calibration target (for instance, a corner on the checkerboard pattern in Figure 5a). When the camera translates, the position of the image of each reference point also changes. On the other hand, measuring rotation consists of observing how a speckle pattern moves on the image plane as the camera rotates. To uncouple translation from rotation, the observation of both the reference point and the speckle pattern must be performed simultaneously. This can be achieved in practice by using the setup shown in Figure 14. In Figure 14a, a speckle field is produced by back illuminating a diffusing material with a laser. Installing a pinhole in front of the diffusing material causes a circular speckle pattern to be observed by the camera as shown in Figure 14b. The source generating this circular speckle pattern is called an *Almost Punctual Speckle Source* (APSS) in the following. The image of the APSS in the subjective speckle configuration of Figure 14b is shown in Figure 14c while the actual assembly of the APSS is shown in Figure 14d. The fact that the source is *almost* punctual is very important since a perfect point source would not generate a speckle pattern on the image plane and speckle could not be used for estimating rotation. In addition to its ability to produce a speckle pattern on the image plane, the APSS has the important feature that it can be used as a *reference point* for measuring *translation*. Indeed, the APSS being close to a point source, the edge of the circle circumscribing the speckle pattern is always in focus, and the geometric center of the circle follows the pinhole image formation model. Consequently, when moving the camera in front of the APSS, the position of the image of the geometric center of the circle is affected by translation and rotation (and can be used as a calibration reference point) while the speckle pattern circumscribed by the circle is affected by rotation only.

However, as seen in Figure 14c, the speckle causes the edge of the circle to be jagged and the distribution of bright and dark areas inside the edge to be irregular. This affects the accuracy of the estimate of the position of the geometric center of the circle. A more accurate estimate of this position would be obtained if the intensity were uniform inside and on the edge of the circle. The desired uniformity can be obtained experimentally by adding a rotating mask made of frosted plexiglass between the APSS and the camera (see Figure 14e). Since the mask rotates at high speed, it causes the speckle pattern to change very rapidly, which in turn causes the image of the pattern to be uniform during acquisition (see Figure 14f). In short, two images are acquired for each camera position: (*i*) an image with the mask in front of the APSS used to estimate the position of the geometric center of the circle and (*ii*) a second image without the mask used to observe the speckle pattern. Image acquisition is triggered electronically by the rotating mechanism, which is mounted close to the APSS but on a different stand so that the vibration caused by the rotating mask does not affect the APSS. The implementation details of the APSS are listed in Table 5.

#### 4.3.3. A Four-Step Procedure for Calibrating $E_{MC}$

**Figure 15:**
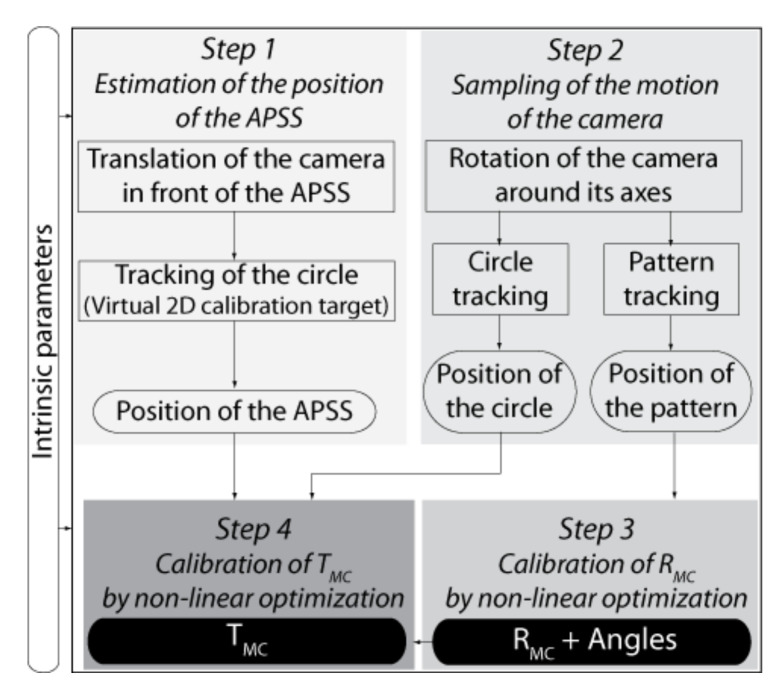
Four-step calibration procedure for the ASP using speckle metrology and the APSS. Step 1 consists of the estimation of the position of the APSS in the reference frame of the camera. Step 2 consists of sampling the motion of the camera by rotating it (one axis at a time) in front of the APSS and recording the images of the speckle circle and speckle pattern at several angular positions. Calibration of the rotation component of transform $E_{MC}$ is performed at Step 3 by optimizing the parameters of the transform for minimizing the reprojection error of the circle and pattern on the image plane of the camera. The translation component of $E_{MC}$ is estimated at Step 4, again by non-linear optimization.

The Four-step procedure for calibrating $E_{MC}$ is shown in Figure 15. In this procedure, it is assumed that the intrinsic parameters of the cameras have been calibrated beforehand (see Section 4.2). The procedure is presented for one camera only since it is similar for both cameras of the ASP.

**STEP 1: Estimation of the Position of the APSS in the Reference Frame of the Camera**

The first step of the procedure for calibrating $E_{MC}$ consists of the estimation of the position $S_{C}$ of the APSS in the reference frame of the camera in its initial configuration (i.e., $\phi_{1}=\phi_{2}=0$ in Figure 6). This can be achieved by (*i*) finding the frame transformation $E_{CW}$ expressing the position and orientation of the APSS in $O_{C}$, the reference frame of the camera, and (*ii*) computing $S_{C}$, the position of the APSS in $O_{C}$ using $E_{CW}$.

**Figure 16:**
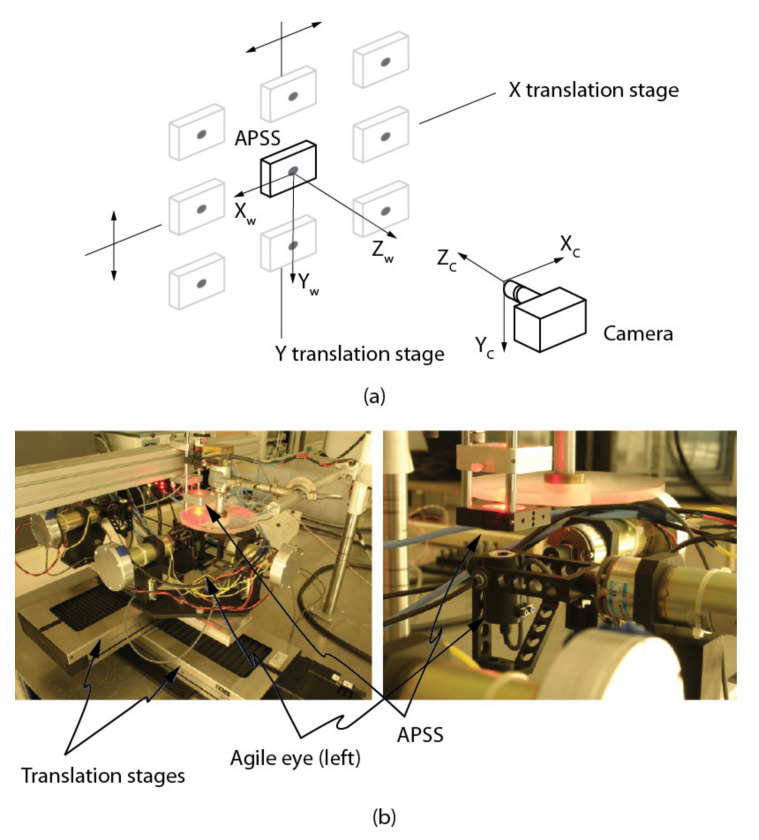
Estimation of the position of the APSS in the reference frame of the camera. The APSS is mounted on two precision linear stages (not shown in the diagram) and is moved at different known locations in frame $O_{W}$. The center of gravity of the image of the circle on the image plane is found at each position. This operation mimics the operation of imaging a calibration target and finding correspondences between image points in the image reference frame and calibration points on the target (**a**). Linear stages, APSS and left eye of the ASP (left); APSS and camera of the left eye of the ASP pointing upward (right) (**b**).

The approach starts by translating the APSS in front of the camera using two precision linear stages mounted at 90°, as shown in Figure 16a. At least four different positions are required. For each position, an image of the APSS is acquired (with the plexiglass moving in front of it, see Figure 16b for details of the experimental setup) and the center of gravity of the circle is computed with sub-pixel accuracy (see Appendix A for details). The group of positions—centers of gravity pairs form a virtual calibration target mimicking the one shown in Figure 5. Assuming the origin $O_{W}$ of the reference frame attached to the “virtual” calibration target is located at the APSS and the orientation of its axes is along the translation stages, the problem of computing $S_{C}$ stands as follows:

(32) $$s\left[\begin{array}{c} u_{i}\\ v_{i}\\ 1 \end{array}\right]=KE_{CW}\left[\begin{array}{c} X_{Wi}\\ Y_{Wi}\\ Z_{Wi}\\ 1 \end{array}\right]$$

where ${\left[\begin{array}{ccc} u_{i} & v_{i} & 1 \end{array}\right]}^{t}$ is the position of the center of gravity of the circle in the *i*th image and ${\left[\begin{array}{cccc} X_{Wi} & Y_{Wi} & Z_{Wi} & 1 \end{array}\right]}^{t}$ is the corresponding position of the camera set by the translation stages. The estimation of $E_{CW}$ is nothing but the procedure described above for the calibration of the pose of a camera with respect to a calibration target. Once $E_{CW}$ is known, the APSS is moved with the translation stages so that the image of the circle is located as much as possible at the center of the field of view on the image plane. $S_{C}$ can then be computed. One way of doing this is to read the values of the translation stages, which provide the position $S_{W}$ of the APSS in frame $O_{W}$, and then to find $S_{C}$ with:

(33) $$S_{C}=E_{CW}S_{W}$$

However, this approach is error-prone due to various measurement errors, which were found to have an adverse effect on the calibration of $E_{MC}$. A better procedure is as follows. Let ${\left[\begin{array}{ccc} u & v & 1 \end{array}\right]}^{t}$ be the coordinates of the center of gravity of the image of the circle on the image plane. The equation of the projector going from this point to the APSS is given by:

(34) $${\vec{v}}_{C}=K^{-1}\left[\begin{array}{c} u\\ v\\ 1 \end{array}\right]$$

where *K* is given by Equation (2). The APSS must be located along this projector and the following thus holds true for a scalar *α*:

(35) $$S_{C}=\alpha {\vec{v}}_{C}$$

Now, the third column of $E_{CW}$ contains the components of the *Z*-axis of the virtual calibration target expressed in frame $O_{C}$:

(36) $${\vec{n}}_{C}=r_{CW3}$$

In addition, $t_{CW}$, the translation component of $E_{CW}$, corresponds to the position of the origin of $O_{W}$ in frame $O_{W}$ and thus provides a point belonging to the virtual calibration plane:

(37) $$P_{C}=t_{CW}$$

Knowing a point and the normal $\left({\vec{n}}_{C}\right)$ to the virtual calibration plane, and knowing that $S_{C}$ also lies on this plane, one can write:

(38) $${\vec{n}}_{C}\;\cdot \;\left(P_{C}-S_{C}\right)=0$$

Replacing Equation (35) in Equation (38) and solving for α gives:

(39) $$\alpha =\frac{{\vec{n}}_{C}\;\cdot \;P_{C}}{{\vec{n}}_{C}\;\cdot \;{\vec{v}}_{C}}$$

This value for α enables the computation of $S_{C}$ with Equation (35) and completes STEP 1.

In the above procedure, it is assumed that the linear translation stages are perfectly perpendicular, a condition that is difficult to meet in experimental conditions. It is possible to take the non-orthogonality between the translation axes into account in the calibration procedure. Doing so improves the quality of the estimation of $E_{CW}$ and, by the same token, the quality of the estimate for $S_{C}$. A method for taking this non-orthogonality into account in the calibration procedure is presented in Appendix C.

**STEP 2: Sampling of the Angular Positions of the Agile Eye**

**Figure 17:**
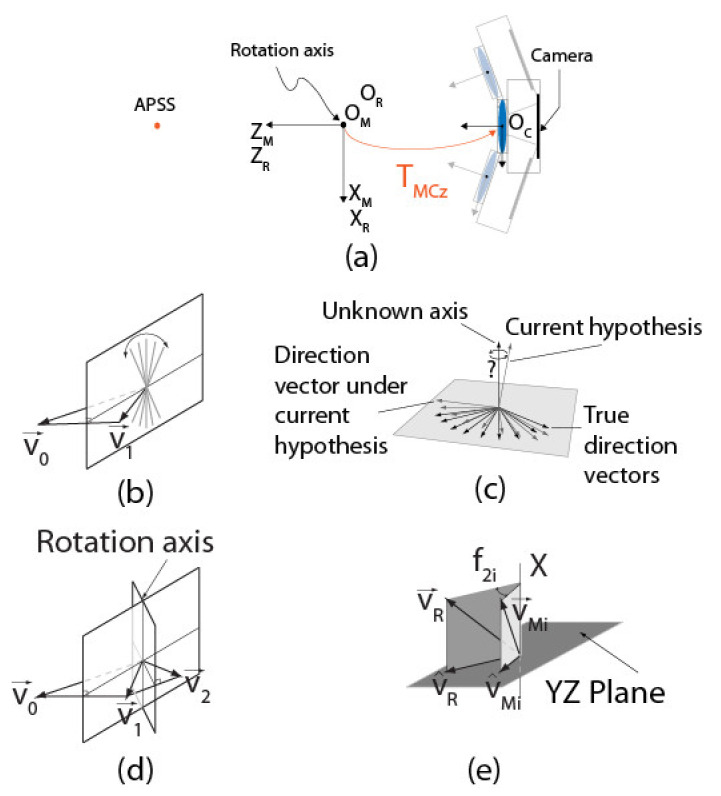
Procedure for calibrating $E_{MC}$. The camera observes the APSS while being positioned at different orientations caused by rotations around the *Y*-axis (longitudinal axis). The observation of the speckle pattern at each position allows the pose of the camera to be estimated with respect to the orientation device ($T_{MCz}$ for this 2D example). The same procedure is applied for the *X*-axis (latitudinal axis) (**a**). Any axis in a plane can explain the rotation from $V_{1}$ to $V_{2}$ (**b**). Unknown axis and current hypothesis in the non-linear optimization algorithm (**c**). Change in the orientation of a direction vector (**d**). Estimation of the rotation angle by projecting the direction vectors on the plane perpendicular to the rotation axis (**e**).

**Figure 18:**
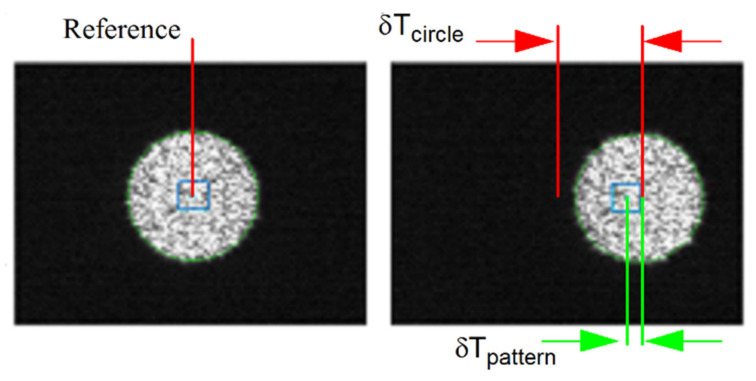
A rotation around axis $O_{M}$ of the manipulator causes the camera to translate and rotate and the image of the APSS to translate by $\delta T_{circle}$. In addition, the rotation causes a speckle point to move inside the image of the APSS by $\delta T_{pattern}$.

Once STEP 1 is complete, the next operation consists of acquiring two sets of images, one for each axis of the camera (this is repeated for both cameras of the ASP). The first series is acquired while the camera executes a longitudinal angular sweeping of the APSS (i.e., around axis Y) and the second one for a latitudinal sweeping of the APSS (around axis X), see Figure 17a for a diagram of the longitudinal sweeping operation). For each sweeping operation around one axis, the other axis is kept at its initial position ($\phi_{1}=0$ or $\phi_{2}=0$ on Figure 6) and the images of the circle (with the diffusing plexiglass in position) and the speckle pattern (with the diffusing plexiglass removed) are stored at each angular position. Since $E_{MC}\neq I$, the rotations will cause the position of the geometric center of the circle to move by $\delta T_{circle}$ (because of the rotation and translation of frame $O_{C}$ in frame $O_{M}$) and the position of the speckle pattern circumscribed by the circle to move by $\delta T_{pattern}$ (because of the rotation of frame $O_{C}$). This behavior is shown in Figure 18.

Once the images are acquired, they are processed for each angular position to find: (*i*) the center of gravity of the circle and (*ii*) the position of the speckle pattern. The approaches for computing these parameters are detailed in Appendix A and Appendix B, respectively. This information is used in Steps 3 and 4 of the calibration procedure.

**STEP 3: Estimation of the Rotation Component of *E_MC_* by Optimization**

STEP 3 aims at estimating $R_{MC}$, the transform representing the *rotation* component of $E_{MC}$, using $\delta T_{pattern,i}$, the displacement of the speckle pattern for each angular position *i* imposed at STEP 2. The strategy consists of finding the orientation of the longitudinal and latitudinal axes of frame $O_{M}$ in frame $O_{C}$, the reference frame of the camera. Knowing the orientation of these axes is all that is needed to estimate $R_{MC}$ since, according to the geometric model of each eye of the ASP (see Figure 4 and Figure 6), the latitudinal and longitudinal rotation axes correspond to axes X and Y of frame $O_{M}$. When the orientations of two orthogonal axes of a reference frame are known in another reference frame, the orientation of the reference frame itself is known. In other words, finding the orientation of the longitudinal and latitudinal axes of frame $O_{M}$ in frame $O_{C}\;$ allows finding $R_{MC}$, the rotation component of frame $O_{M}$ in frame $O_{C}$. $R_{MC}$ is simply obtained as $R_{CM}^{-1}$. Strictly speaking, the latitudinal and longitudinal axes found at STEP 3 are axes X and Y of frame $O_{R}$ in frame $O_{C}$ since these axes are the ones that are actuated by the motors; however, under the experimental conditions described at STEP 2, which impose that the rotations around each axis are performed when the other axis is kept in its initial position $\left(\phi_{1}=0\;\mathrm{or}\;\phi_{2}=0\right)$, frame $O_{M}$ is superimposed perfectly with frame $O_{R}$ and the axes found are also those of frame $O_{M}$ in frame $O_{C}$.

Before describing the non-linear optimization algorithm that is used for finding the orientation of the latitudinal and longitudinal axes of $O_{M}$ in $O_{C}$, a geometric interpretation of the procedure and its relationship with the image acquisition process described at STEP 2 are presented first. Finding the axes (and their orientation) is achieved by imposing rotations of frame $O_{C}$ in frame $O_{M}$. A single rotation is not enough since a rotation around any axis lying on a plane normal to the line joining the two vectors can bring a direction vector ${\vec{V}}_{0}$ to a new position ${\vec{V}}_{1}$ as shown in Figure 17b).

However, only one axis can bring ${\vec{V}}_{0}$ to ${\vec{V}}_{1}$ and ${\vec{V}}_{2}$ (see Figure 17d) with two successive rotations. It is why, at STEP 2, a longitudinal sweeping of the camera in front of the APSS is executed with the other axis in its initial position. The different angular positions $\theta_{i}$ (with i>2) allow a direction vector ${\vec{V}}_{0}$ corresponding to a speckle point in the first image of the sweep to be brought to different directions ${\vec{V}}_{i}\left(\theta_{i}\right)$ corresponding to the same speckle point that has moved by $\delta T_{pattern,i}$ due to the rotation (Figure 13a,b). One way of finding the longitudinal axis (and latitudinal axis afterwards) would be to compute the intersection of the plane bringing ${\vec{V}}_{0}$ on ${\vec{V}}_{1}$ and the plane bringing ${\vec{V}}_{1}$ on ${\vec{V}}_{2}$ (and repeat this for all pairs of direction vectors ${\vec{V}}_{i-1}$ on ${\vec{V}}_{i}$ and ${\vec{V}}_{i}$ on ${\vec{V}}_{i+1}$).

However, this approach was found to be sensitive to measurement noise. A much more accurate method consists of using non-linear optimization with a cost function directly linked to observations in the image (i.e., the speckle pattern and associated direction vectors). As shown in Figure 17c, the non-linear optimization method formulates a hypothesis for the axis to be estimated and computes the angular displacements using this axis and the displacement of the speckle pattern from one image to the other. Using the hypothesis for the axis and the computed angular displacements, it is possible to find the estimates of direction vectors. Since ${\hat{V}}_{i}$, the hypothesis for the axis may initially be far from the true axis, vectors ${\hat{V}}_{i}$ do not correspond to the true motion of the speckle pattern. The cost function uses these estimates and the direction vectors ${\vec{V}}_{i}$ measured in the images to generate a new estimate for the rotation axis until convergence. This procedure is now described formally.

**Figure 19:**
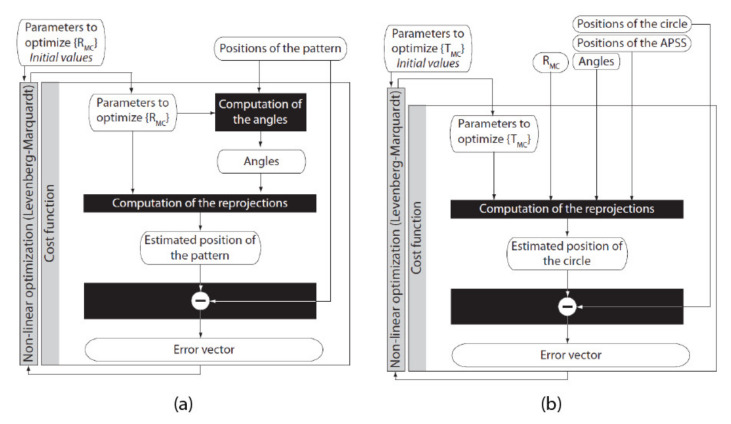
Details of the procedure for estimating $R_{MC}$, the rotation component of $E_{MC}$ (**a**). Once $R_{MC}$ is found, it is used for estimating $T_{MC}$, the translation component of $E_{MC}$ (**b**). These procedures correspond to Steps 3 and 4 in Figure 15.

The non-linear optimization procedure presented in Figure 19a is used for estimating the orientation of the longitudinal and latitudinal axes. As shown in the figure, the rotation angles that were imposed on the agile eye at STEP 2 are estimated. Each rotation axis (latitudinal and longitudinal) is processed separately. A direction vector ${\vec{v}}_{ci}$ (see Figure 13c can be computed for each angular position of the camera. This vector is expressed in the reference frame of the camera (i.e., $O_{C}$). The coordinates $\left(u_{i},v_{i}\right)$ of the reference point on the speckle pattern from which the vector originates can be expressed as coordinates on the normalized image plane with:

(40) $$\left[\begin{array}{c} x_{i}\\ y_{i}\\ 1 \end{array}\right]=K^{-1}\left[\begin{array}{c} u_{i}\\ v_{i}\\ 1 \end{array}\right]$$

where $K^{-1}$ is the inverse of K in Equation (2). The components of the direction vector are obtained with the following equation (since this vector passes through the origin of the camera reference frame):

(41) $${\vec{v}}_{Ci}=\left[\begin{array}{c} x_{i}\\ y_{i}\\ 1 \end{array}\right]-\left[\begin{array}{c} 0\\ 0\\ 0 \end{array}\right]=\left[\begin{array}{c} x_{i}\\ y_{i}\\ 1 \end{array}\right]$$

The point on the speckle pattern being used as a reference and being tracked at each angular position corresponds to direction vector ${\vec{v}}_{R}$ expressed in $O_{R}$, a fixed reference frame. According to the model of the agile eye (Figure 4, left or right eye with $O_{M}$ superimposed with $O_{R}$), the relation between ${\vec{v}}_{Ci}$ and ${\vec{v}}_{R}$ is given by (only rotations are considered here):

(42) $${\vec{v}}_{R}=R_{RMi}R_{MC}{\vec{v}}_{Ci}$$

Although the matrix to be calibrated is $R_{MC}$, the key matrix in Equation (42) is $R_{RMi}$ since it is through this matrix that the camera is rotated because it models the parallel mechanism; however, $R_{RMi}$ is unknown. As a matter of fact, in the optimization loop of Figure 19a, $R_{MC}$ is assumed to be known and each iteration aims at refining this estimate as described next.

The focus on $R_{RMi}$ is expressed explicitly by rewriting Equation (42):

(43) $${\vec{v}}_{R}=R_{RMi}R_{MC}{\vec{v}}_{Mi}$$

with:

(44) $${\vec{v}}_{Mi}=R_{MC}{\vec{v}}_{Ci}$$

In Equation (44), ${\vec{v}}_{Mi}$ can be computed directly since $R_{MC}$ (and its inverse $R_{CM}^{-1}$), is assumed to be known. The reference direction vector ${\vec{v}}_{R}$ can also be computed with Equation (43) by considering that it is ${\vec{v}}_{C0}$, the direction vector for angular position i=0 which leads to:

(45) $${\vec{v}}_{R}=R_{RM0}R_{MC}{\vec{v}}_{C0}$$

In the initial configuration (i.e., i=0, $\phi_{1}=\phi_{2}=0$), $R_{RM0}=I$ and ${\vec{v}}_{C0}$ can be computed directly with the hypothesis for $R_{MC}$.

All that remains is to find matrices $R_{RMi}$ allowing us to bring ${\vec{v}}_{Mi}$ on ${\vec{v}}_{R}$ and find the angles associated with each rotation of the agile eye. Based on the model of the agile eye and Equations (13) and (12), matrix $R_{RMi}$ is given by (for each camera):

(46) $$R_{RMi}=R_{RiMi,y}R_{RiMi,x}$$

Strictly speaking, it is not possible to find $R_{RMi}$ because only one equation (Equation (43)) is available to compute two unknowns ($\phi_{1}$ and $\phi_{2}$). This explains why each axis was processed separately at STEP 2. Let us assume that only the rotations around the latitudinal axis are considered. In this case, X is the rotation axis and $\phi_{1}=0$ since the rotation around Y is null. This leaves only one unknown that is computed with Equation (43). Projecting ${\vec{v}}_{Mi}$ and ${\vec{v}}_{R}$ on plane YZ, which is normal to axis X as shown in Figure 17d, yields ${\bar{v}}_{Mi}$ and ${\bar{v}}_{R}$. The angle between the projections can be found from their scalar product:

(47) $${\bar{v}}_{R}\cdot {\bar{v}}_{Mi}=∥{\bar{v}}_{R}∥∥{\bar{v}}_{Mi}∥\cos\phi_{2i}$$

The value found for $\phi_{2i}$ can be used in Equation (46) to find $R_{RMi}$. The procedure is similar for rotations around the longitudinal axis (Y). Returning to Figure 19a, the next operation consists of computing the reprojections of the direction vectors on the image plane using the current hypothesis for $R_{MC}$, angles $\phi_{2i}$, and matrices $R_{RMi}$. This starts by simulating the rotations of the agile eye for estimating the orientations of direction vectors in the reference frame of the camera with:

(48) $${\hat{v}}_{Ci}=R_{MC}^{-1}R_{RMi}^{-1}{\vec{v}}_{R}$$

Direction vector ${\hat{v}}_{Ci}$ is a computation of the direction vector while ${\vec{v}}_{Ci}$ represents a measurement of the direction vector from the observation of the speckle pattern. When the optimization process is initiated, the hypothesis for $R_{MC}$ is likely to be far from its actual value and ${\hat{v}}_{Ci}$ and ${\vec{v}}_{Ci}$ are different. In Equation (48), ${\hat{v}}_{R}$ is given by Equation (45). The projection ${\hat{m}}_{i}$ of ${\hat{v}}_{Ci}$ on the image plane is given by:

(49) $${\hat{m}}_{i}≅\left[\begin{array}{c} s{\hat{u}}_{i}\\ s{\hat{v}}_{i}\\ s \end{array}\right]=K{\hat{v}}_{Ci}$$

while the projection $m_{i}$ of ${\vec{v}}_{Ci}$ measured on the image plane is:

(50) $$m_{i}≅\left[\begin{array}{c} su_{i}\\ sv_{i}\\ s \end{array}\right]=K{\vec{v}}_{Ci}$$

If $M_{a}$ rotations are sampled for axis *a*, the error vector between computed values and measured values of direction vectors is defined as:

(51) $$\xi_{a}=\left[\begin{array}{cc} \left(m_{u1}-{\hat{m}}_{u1}\right) & \left(m_{v1}-{\hat{m}}_{v1}\right) \end{array}\cdots \begin{array}{cc} \left(m_{uM}-{\hat{m}}_{uM}\right) & \left(m_{vM}-{\hat{m}}_{vM}\right) \end{array}\right]$$

The Levenberg–Marquardt non-linear optimization algorithm uses this error vector to minimize the following cost function:

(52) $$min_{\Omega }=\underset{a=\left(X,Y\right)}{\sum }\overset{M_{a}}{\underset{i=1}{\sum }}∥m_{i}-{\hat{m}}_{i}∥{}^{2}$$

where $\Omega =\left\{\alpha ,\beta ,\gamma \right\}$ are the parameters defining $R_{MC}$, $m_{i}$ is the projection of the direction vector of the speckle pattern measured for camera angular position *i*, $M_{a}$ is the number of sampled angular positions for axis *a*, ${\hat{m}}_{i}=Proj\left(K,\Omega ,m_{j},m_{i}\right)$ is the projection of the direction vector of the speckle pattern computed with the current hypothesis for $R_{MC}\left(\Omega \right)$, and $m_{j}$ is the projection of the reference direction vector.

In the preceding procedure, data collected from rotations around each axis have been processed independently up to Equation (51). The results related to each axis need to be combined in the cost function described by Equation (52) to optimize all parameters of $R_{MC}$ simultaneously. Doing so instead of estimating the orientation of each axis of rotation independently makes sure that the orthogonality constraint between the two rotation axes is met.

**STEP 4: Estimation of the Translation Component of** $E_{MC}$ **by Optimization**

The last step for the calibration of $E_{MC}$ is to estimate the translation component $T_{MC}$ of the transform by non-linear optimization. The procedure for calibrating $T_{MC}$ is described in Figure 19b. It accepts as inputs: (*i*) the position of the APSS in the reference frame of the camera found at STEP 1, (*ii*) the position of the center of gravity of the circles (sampled at STEP 2 and computed with the approach described in Appendix A for different angular positions of the camera), (*iii*) $R_{MC}$ found at STEP 3, (*iv*) initial values for the three elements of $T_{MC}$, and (*v*) the values of the angles for the angular positions of the camera.

The procedure for computing the values of the angles for the angular positions of the camera is the one that was used in the non-linear optimization algorithm for finding $R_{MC}$ at STEP 3 (using Equations (42)–(47), but this time, the estimate for $R_{MC}$ is used).

The above information is fed to a Levenberg–Marquardt non-linear optimization algorithm, which now refines the translation parameters of $T_{MC}$ using the measured and computed reprojections of the center of gravity of the circles for each angular position. The reprojections are computed as follows. The position of the APSS in frame $O_{R}$ is:

(53) $${\tilde{S}}_{R}=E_{R0M0}E_{MC}{\tilde{S}}_{C0}$$

In Equation (53), $E_{R0M0}=I$ since the camera is at its initial angular position. We thus have

(54) $$E_{MC}=R_{MC}T_{MC}$$

where $R_{MC}$ is the matrix found at STEP 3 and $T_{MC}$ is assumed to be known since it is the hypothesis. With ${\tilde{S}}_{R}$ known, the position of the source in the reference frame of the camera for each angular position is given by:

(55) $${\tilde{S}}_{Ci}=E_{MC}^{-1}E_{RiMi}^{-1}{\tilde{S}}_{R}$$

with $E_{RiMi}$ given by Equation (11). Again, each axis is processed independently, which means that either $\phi_{1}=0$ or $\phi_{2}=0$. Finally, the coordinates of the reprojected center of gravity of the circle on the image plane are given by:

(56) $${\hat{p}}_{i}≅\left[\begin{array}{c} s{\tilde{u}}_{i}\\ s{\tilde{v}}_{i}\\ s \end{array}\right]=K{\tilde{S}}_{Ci}$$

The error vector for axis *a* is given by:

(57) $$\xi_{a}=\left[\begin{array}{cc} \left(p_{u1}-{\hat{p}}_{u1}\right) & \left(p_{v1}-{\hat{p}}_{v1}\right) \end{array}\cdots \begin{array}{cc} \left(p_{uM}-{\hat{p}}_{uM}\right) & \left(p_{vM}-{\hat{p}}_{vM}\right) \end{array}\right]$$

and is used to minimize:

(58) $$min_{\Omega }=\underset{a=\left(X,Y\right)}{\sum }\overset{M_{a}}{\underset{i=1}{\sum }}∥p_{i}-{\hat{p}}_{i}∥{}^{2}$$

where $\Omega =\left\{T_{MCx},T_{MCy},T_{MCz}\right\}$. In Equation (58), $p_{i}$ is the position of the projection of the center of gravity of the circle being observed at angular position *i*, $M_{a}$ is the number of angular positions of the camera, and ${\hat{p}}_{i}=Proj\left(K,R_{MC},\Omega ,S_{Ci},\varphi_{ai}\right)$ is the projection of the center of gravity of the circle computed with the current hypothesis for $T_{MC}$.

The significant advantage of the calibration approach using speckle metrology is that it uncouples the estimation of $R_{MC}$ and $T_{MC}$ completely since different (and independent) image information operations (displacement of the speckle pattern for $R_{MC}$ and displacement of the center of gravity for $T_{MC}$) are used to estimate each transform. Consequently, a small rotation cannot be confused for a small translation and vice-versa.

## 5. Experiments

**Figure 20:**
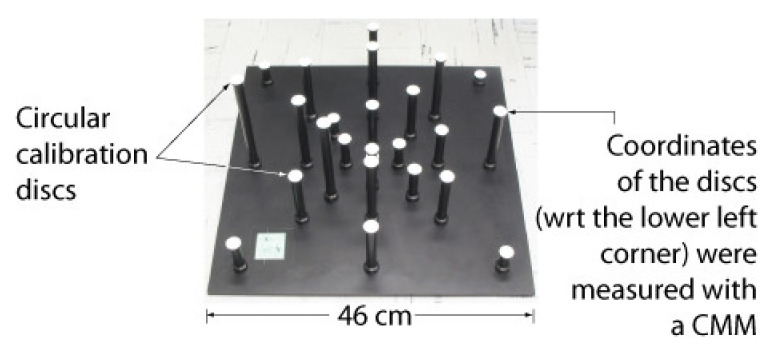
The 3D calibration target used for the stereo experiments. The target is circumscribed in a 500 mm × 500 mm × 250 mm box. The coordinates of the center of the white discs have been measured with a high precision CMM and are used as ground truth.

A series of four experiments were conducted to validate the calibration approach based on speckle metrology. The first experiment aimed at validating the use of speckle for measuring rotations (i.e., angular measurements). This experiment was performed separately on the two eyes of the ASP to make sure that the approach was reproducible and did not depend on a specific mechanism. The second experiment aimed at calibrating $E_{MC}$ using the four-step procedure in Section 4.3. Again, the experiment was repeated independently for each eye. For this experiment, both eyes were set in a one-axis configuration, meaning that only the longitudinal axis (*Y*-axis) was used while the latitudinal axis remained motionless. The third experiment used the calibrated ASP (one-axis configuration) in real stereo measurement conditions. The 3D positions of the white circular discs of the calibration target shown in Figure 20 were measured using the ASP and the accuracy of the measurements was compared to ground truth data obtained with a Coordinate Measuring Machine (CMM).

Finally, the fourth experiment focused on the calibration of a two-axis configuration (i.e., both the longitudinal and latitudinal axes in operation). This experiment not only helped to validate the speckle-based calibration approach on the full system, but also to observe a previously unknown behavior of the parallel mechanism.

Before performing the above experiments, two preliminary operations need to be executed. The first one consists of setting the focus of the cameras at infinity, a condition that is needed to make the speckle pattern insensitive to translation. The second operation consists of calibrating the intrinsic parameters of the cameras using Zhang’s method [25].

### 5.1. Experiment 1: Measurement of Angular Displacements Using Laser Speckle

In this experiment, the use of the displacement of the speckle pattern is validated as a means of estimating the rotations of a camera. This validation is important since the displacement of the speckle pattern under rotation is the phenomenon on which is based the calibration of the ASP or any stereo pair mounted on tilt/pan mechanisms. In this experiment, it is important to mention that radial lens distortion is not accounted for in the measurement of the displacement of both the speckle pattern and the circle. The reason is that, as illustrated in Figure 14, the APSS is observed out-of-focus when images of the speckle pattern and the circle are sampled, although the experiment is run with the camera focus set at infinity. The parameters modeling radial lens distortion were estimated with Zhang’s method using a standard target (Figure 5) observed in focus; they are useless in different imaging conditions.

The experiment consists of rotating the camera around its longitudinal axis when observing the APSS. The displacement of the speckle pattern observed during rotation is used to estimate the rotation angles, a key operation at STEP 3 of the calibration of *E_MC_* (Figure 19a).

**Figure 21:**
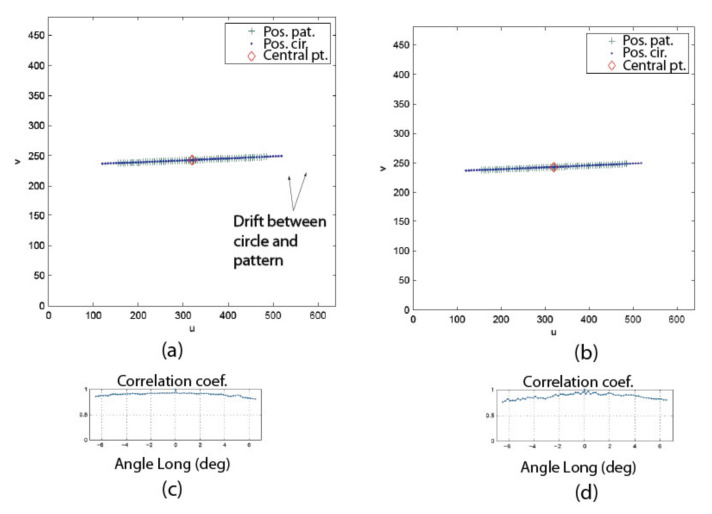
Position of the pattern and the circle observed when the camera of the left (**a**) and right (**b**) eyes rotate around their longitudinal axis. Value of the correlation coefficient for speckle pattern detection for the left eye (**c**) and right (**d**) eye.

The behavior of the pattern is validated by analyzing the reprojection error of the speckle pattern as well as the error on the angular measurements. Table 6 lists the values of the parameters for the experiment. Figure 21 shows the first set of results. The displacements of the speckle pattern and the circle in the image for the left (a) and right (b) eyes are shown for the experimental conditions described in Table 6. It is interesting to observe the drift in position between the pattern and the circle during rotation. For instance, the last blue dot on the right-hand side of Figure 21a corresponds to the last green cross but is located at a larger *u* coordinate on the image plane. This is proof that the center of projection of the camera is not located on the rotation axis and thus undergoes both translation and rotation (the displacement of the speckle pattern being insensitive to translations, it is moved less far from the origin on the *u* axis than the circle). It is also proof that the observation of an APSS uncouples translation from rotation. Figure 21c,d show that the correlation coefficient remains high on the whole rotation interval and that the confidence in the estimated position of the speckle pattern is also high.

**Figure 22:**
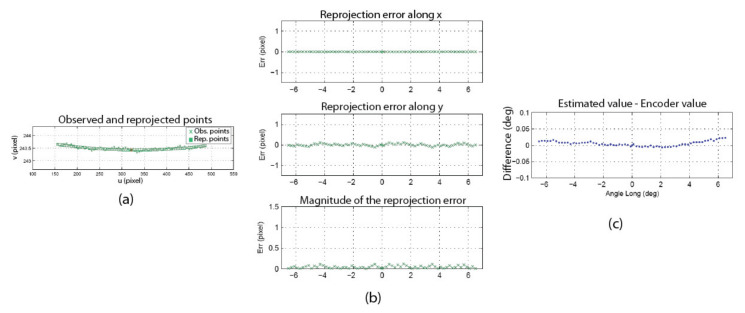
Observed speckle points and reprojected points obtained from the estimation of the axes and rotation angles (**a**). Reprojection error along X and Y in the image plane and magnitude of the reprojection error (**b**). Comparison of the estimated values of the rotation angles vs. the value read on the encoders on the axis (**c**). These plots are for the left eye.

The information on the position of the circle and the position of the speckle pattern in Figure 21 is processed as described in Section 4.3 (STEP 3) to find the angular position of the camera as well as the orientation of the longitudinal axis of the camera in frame $O_{M}$ (in this experiment, only the longitudinal axis is computed since the system is in a one-axis configuration). Figure 22 shows the result of these computations for the left eye of the ASP. Similar results were obtained for the right eye. Figure 22a shows that the positions of the speckle pattern (expressed as direction vectors corresponding to the different positions of a speckle point being tracked) reprojected on the image plane using the model for the estimation of the axes and rotations are very close to the actual measurements. Figure 22b shows quantitative results with plots of the reprojection error for the X and Y axes of the image plane, as well as the magnitude of the error. This error is almost always smaller than 0.1 pixel (which is below the level of precision of the method used for tracking the speckle pattern).

These results validate several aspects of the calibration method. Firstly, they confirm the hypothesis on the behavior of the speckle pattern when the camera rotates in front of the APSS and that the camera pinhole model is adequate for supporting the calibration procedure. The results also confirm the efficiency of the non-linear optimization procedure for computing the orientation of the axes. Finally, the accuracy of the approach for computing the rotation angles is demonstrated in Figure 22c, which plots the difference between estimated values of the angles and the values of the angles read on the encoders mounted on the axes of the parallel mechanisms (Figure 1b). These encoders are not used in the calibration process but are only used for real-time control of the motors. The results show that this difference does not exceed 0.05° on the angular interval defined in Table 6.

### 5.2. Experiment 2: Calibration of $E_{MC}$ for a One-Axis Configuration of the Cameras of the ASP

The second experiment presents the results for the calibration of transform $E_{MC}$ using the procedure illustrated in Figure 15. A preliminary step must be executed before calibrating the transform (in addition to the adjustment of the focus at infinity and the estimation of the intrinsic parameters with Zhang’s algorithm). This step consists of adjusting the distance between the camera and the APSS because it has a direct effect on the size of the image of the circle (and the speckle pattern inside its boundary). The image of the circle must remain entirely visible on the whole angular interval mentioned in Table 6 since its center of gravity is used in the calibration process. Consequently, the size of the image of the circle must be kept to a minimum; however, this size must be large enough so the image of the speckle pattern remains within the circles’ boundary for the whole angular interval and rotations can be estimated accurately. For this experiment, the APSS was positioned in front of the camera such that the diameter of the image of the circle was 200 pixels in diameter. This size is large compared to the size of the correlation window (41 × 41 pixels) used for tracking the speckle pattern and allows for accurate detection of the center of gravity of the circle and tracking of the speckle pattern on the angular interval for sampling rotation.

**Figure 23:**
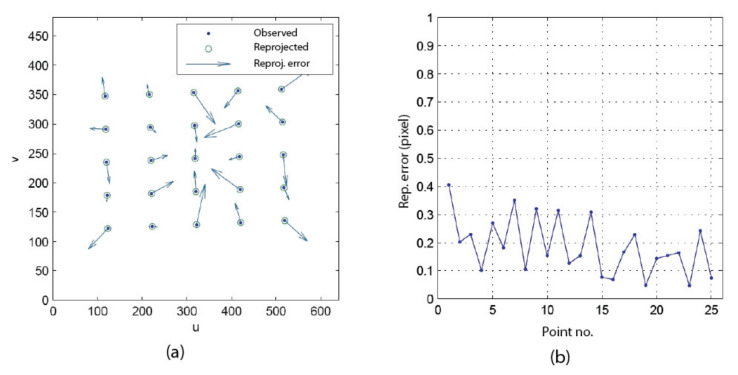
Result of the estimation of the pose of the virtual calibration target in the reference frame of the camera. The dots represent the observed position of 25 calibration points. The circles represent the position of the reprojection of the calibration points using the estimated pose. Since the error is very small, the arrows show the modulus and orientation of the reprojection error with an amplification factor (**a**). Quantitative value of the reprojection error for the 25 calibration points numbered from the right to left/top to bottom. The error is always smaller than 0.4 pixel (**b**).

Figure 23 shows the result of STEP 1 in Figure 15, which consists of finding the position of the APSS in the reference frame of the camera (i.e., finding transform $E_{MC}$ and then finding $S_{C}$ with Equations (35) and (39)). The observed points are shown as dots, while the reprojected points are shown as circles in Figure 23a. Since the reprojection error is very small (under 0.5 pixels on the plot in Figure 23b, its modulus and orientation are plotted in Figure 23a with an amplification factor and show that the direction of the error is random. The non-orthogonality of the translation stages is accounted for in the experiment (see Appendix C). Once $E_{CW}$ is estimated, the APSS is placed in front of the camera and its position in $O_{C}$ is computed with Equation (39). The position found for Experiment 2 was [0.322, −0.164, 20.909]^T^. The accuracy of this estimate is assessed at STEP 4.

**Figure 24:**
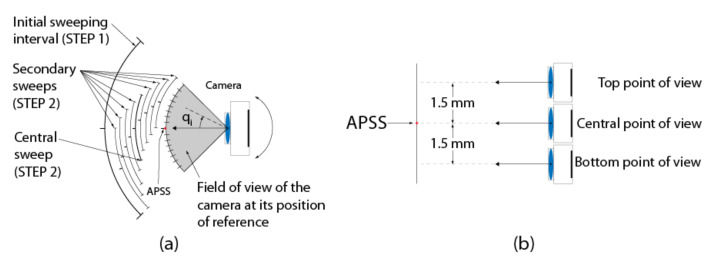
Secondary sweeps for tracking the speckle pattern. Top view (**a**). Side view (**b**).

The second step consists of sampling the angular position of the camera at different angles (Figure 17a). Three improvements are brought to the tracking of the speckle pattern compared to the description given at STEP 2 in Section 4.3. Firstly, instead of sampling the motion of the camera with a single sweep in front of the APSS as explained in Section 4.3, the sweeping movement is repeated from three different points of view as illustrated in Figure 24b. STEP 1 needs to be repeated for each point of view since the position of the APSS must be known to process the set of data collected from each sweep. The next two improvements do not need any additional acquisition and are related to the tracking of the speckle pattern. For the first one, the pattern is tracked within eight secondary sweeps (in addition to the central sweep), as illustrated in Figure 24a. Each secondary sweep covers an angular interval of 6 and the reference position (i.e., angular value of 0° marked with a tick in Figure 24a) between the sweeps is 0.8° within the global calibration interval. The last improvement consists of tracking 25 speckle points simultaneously instead of one. Tracked points are arranged in a 5 × 5 grid centered on the central point (located at the center of the circle for angular position 0°). Speckle points are spread 20 *pixels* apart on the rows and columns. The rationale for using additional points of view, secondary sweeps, and additional speckle points is to increase the robustness of the estimate for $R_{MC}$ (and, by the same token, for $T_{MC}$) to acquisition noise by (*i*) using more observations of speckle points on the whole image plane, (*ii*) include variability in the way the APSS is observed by the camera, and (*iii*) improve the quality of the correlation coefficient by making sure that the tracking window does not come too close to the border of the circle.

**Figure 25:**
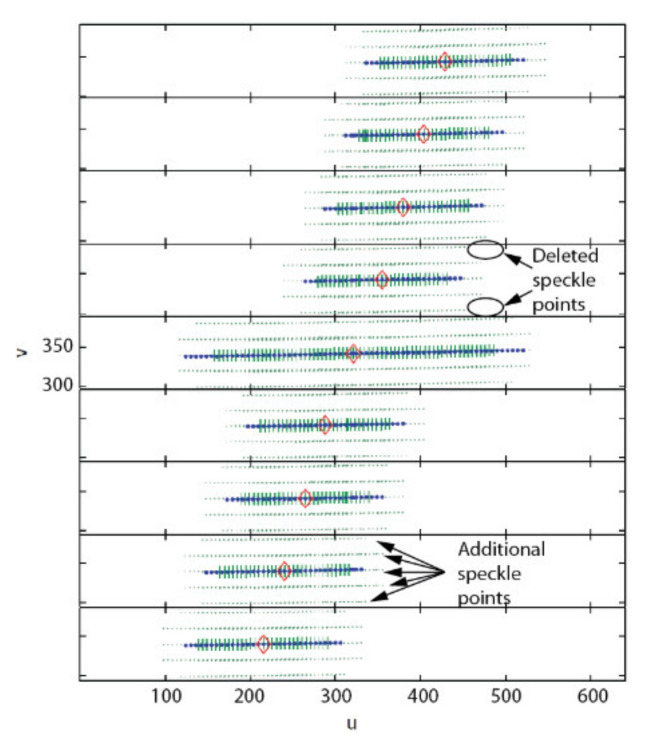
On the left: result of the tracking for eight secondary sweeps and the central sweep, including additional speckle points for the top point of view of the right eye of the ASP. The secondary sweeps are plotted one above the other to reveal the details. In practice, they all overlap the central sweep. Right: magnification of the extremity of a secondary sweep showing missing speckle points dropped from the calibration data because the correlation coefficient was too small (the tracked speckle points were near the border of the circle).

Figure 25 presents the result of the nine sweeps (central +8 secondary) of the angular interval for the top point of view of the right eye of the ASP (results for the left eye are similar). A total of 21 speckle points were tracked for each sweep. As shown in the magnification on the right-hand side of the figure, some speckle points (in this case, four) were dropped from secondary sweeps because the correlation coefficient was too small. Dropped points were not used in the non-linear optimization process leading to the estimation of $E_{MC}$. Figure 25 demonstrates that the behavior of the 27 combinations of speckle pattern and circle for secondary sweeps is very similar to the one of the central sweep, which is predictable since the purpose of the secondary sweeps is to bring redundancy in the data for non-linear optimization. An additional observation is that the line corresponding to each sweep is not perfectly horizontal, which means that $R_{MCz}$, the rotation component of $E_{MC}$ around the optical axis Z, is small but not null. Again, the drift between the position of the circle and the position of the speckle pattern demonstrates that the camera reference frame is not perfectly aligned with frame $O_{M}$ and thus undergoes both rotation *and* translation when the parallel mechanism rotates around its longitudinal axis.

**Figure 26:**
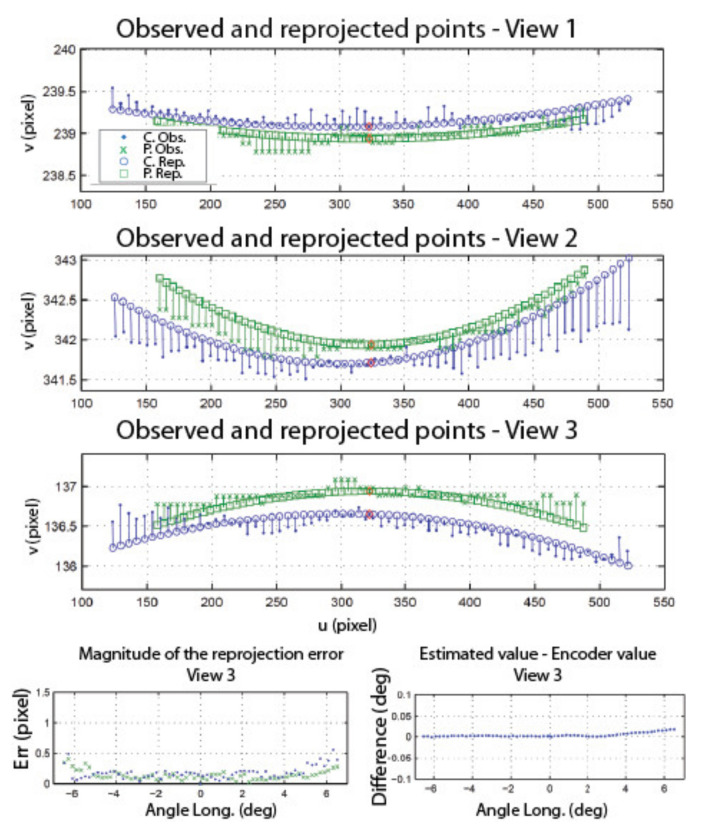
Observed position of the circle and speckle pattern and reprojected circle and speckle pattern based on the estimates for $R_{MC}$ and $T_{MC}$. The central sweep for the three views is shown. The magnitude of the reprojection error for the circle and pattern is under 0.5 pixel and the error in angle measurement is very close to 0°. For display purposes, the small rotation around the optical axis of the camera (component $E_{MCz}$ of $E_{MC}$ in Figure 25) has been corrected.

Once the sweeping of the camera in front of the APSS is complete and the data on the position of the circle and the speckle pattern is acquired, the non-linear optimization process for estimating $R_{MC}$ (STEP 3) and $T_{MC}$ (STEP 4) is executed. Figure 26 presents the *observed* position of the center of gravity of the circle and direction vectors of the speckle pattern and the *reprojected* position of the center of gravity of the circle and the direction vectors of the speckle pattern using the camera model based on the estimated transformations $R_{MC}$ and $T_{MC}$. Due to space limitations, only the central sweep is shown for the three views. Results are similar for the eight secondary sweeps. An important result that demonstrates the validity of the calibration approach is that the reprojection error (bottom left plot in Figure 26) is always smaller than 1 *pixel* for both the circle and the speckle pattern, meaning that the estimates for $R_{MC}$ and $T_{MC}$ are accurate. In addition, the estimates of the rotation angles (bottom right plot in Figure 26) are very accurate, and the error is close to 0° (as a matter of fact, it is approximately 0.004° on the entire angular interval and reaches 0.017° at the right end of the interval).

The three plots at the top center of Figure 26 are interesting for many reasons. Firstly, the reprojection error is small for both the speckle pattern and the circle (bottom left in Figure 26). Secondly, the curves on which the reprojected points lie are slightly different from the curves of the observed points for both the circle and the speckle pattern. This behavior is not limited to the experiment reported in this paper but was observed for all experiments conducted on the left and right eye of the ASP. This suggests that systematic errors affect the calibration procedure. It is suspected that these errors are caused by elements that are *not* accounted for by the camera model and the global model of the ASP; however, since this systematic error has the same order of magnitude as the accuracy of the measurements, it does not invalidate the calibration approach. The experiment described above was repeated for the left eye of the ASP and achieved similar results with respect to the reprojection error of the circle and speckle pattern, and the error on the estimation of the angles. Finally, the stability and precision of the calibration technique were assessed by performing three independent calibration experiments. By independent, it is meant that each calibration was repeated from scratch each time. Table 7 shows the results for the left eye. These results demonstrate that the approach is stable since the values of the parameters for each experiment are close to those of the other experiments and that the reprojection error is approximately 0.25 pixels. Although three experiments do not form a statistically significant sample, they demonstrate the convergence of the non-linear optimization approach towards stable values.

### 5.3. Experiment 3: Validation of the Calibrated Parameters in the Context of a Real Stereo Reconstruction Experiment

The third experiment aims at verifying the accuracy of the parameters calibrated with the speckle metrology approach. By “accuracy”, it is meant that the calibrated parameters can reproduce a real physical situation, namely the pose of the rotation axis of the parallel mechanism relative to the camera. Considering that it is impossible to measure these parameters directly, their precision must be estimated by indirect means. The validation approach that has been selected consists of using the ASP to measure the 3D coordinates of points on an object of known dimensions. This object is the calibration target shown in Figure 20. Since the ASP uses transform $E_{MC}$ of each eye for reconstructing the object, the quality of the reconstruction is an indirect measurement of the quality of the calibration. For the validation experiment to be conclusive for an *active* stereo pair, three tests were performed and consisted of measuring the calibration object for different baselines and with the eyes moving. Three validation tests are presented in the following.

**Figure 27:**
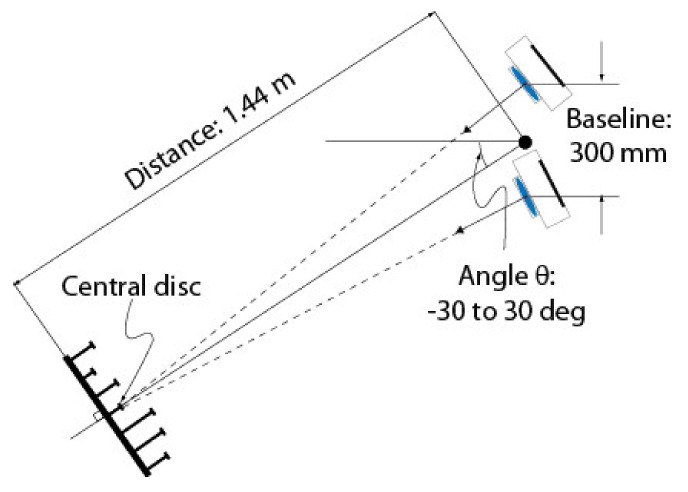
Experimental setup for validating the calibration parameters by 3D reconstruction of a 3D target of known dimensions.

The first validation test consists of reconstructing the 3D target in Figure 20 with the ASP in different configurations. For each configuration, the 3D target is reconstructed from a single stereo view. Overall, the 3D target is reconstructed from 11 directions of observation. As shown in Figure 27, the 11 directions of observation vary between −30° and +30°. The baseline used in this experiment is 300 mm. For each configuration, the distance between the center of the ASP and the central disc of the calibration target is kept constant (based on the experiments, the distance was set to 1.437 m with a standard deviation of 0.009 m). In addition, the experimental procedure made sure that the target was always perpendicular to the line joining the central disc and the center of the ASP. Experiments have demonstrated that this orientation was maintained within 1.57° from the normal direction (standard deviation of 0.72°). To make sure that the larger number of discs were visible from each configuration, each eye of the ASP was adjusted so its optical axis pointed in the direction of the central disc.

The criterion used for assessing the quality of the calibration is the RMS reconstruction error of the 3D target. This error is obtained by comparing the model of the target measured with a CMM with the one measured with the ASP. The RMS error is computed with Equation (59):

(59) $$\epsilon_{RMS}={\left[\frac{1}{N}\overset{N}{\underset{i=1}{\sum }}∥M_{i}-{\hat{M}}_{i}∥{}^{2}\right]}^{1/2}$$

where *N* is the number of discs visible for a given viewpoint, $M_{i}$ is the coordinates of point *i* measured with the CMM, ${\hat{M}}_{i}$ is the coordinates of point *i* reconstructed with the ASP after registration. Registration is the process of bringing two sets of points that are originally expressed in two different coordinate frames into a common one so they can be compared. For the present case, target points measured by the ASP are originally expressed in frame $O_{R_{R}}$ while target points measured by the CMM are expressed in frame $O_{W}$. Registration aims at transforming the points measured by the ASP from frame $O_{R_{R}}$ to frame $O_{W}$. The procedure is performed in two steps. The rigid transform between frame $O_{R_{R}}$ and frame $O_{W}$ is first estimated using an optimization algorithm minimizing the RMS reconstruction error. Once this transform is obtained, it is used to transform points in frame $O_{R_{R}}$ to frame $O_{W}$ in a second step. The RMS error is then computed with Equation (59). The RMS reconstruction error is thus used both as an optimization parameter for the registration step and as a measure of the quality of reconstruction. One may argue that this procedure could bias the interpretation of the results. However, this was accounted for when analyzing the results, and a third validation test has also been designed to minimize the impact of registration on the measurement error.

**Figure 28:**
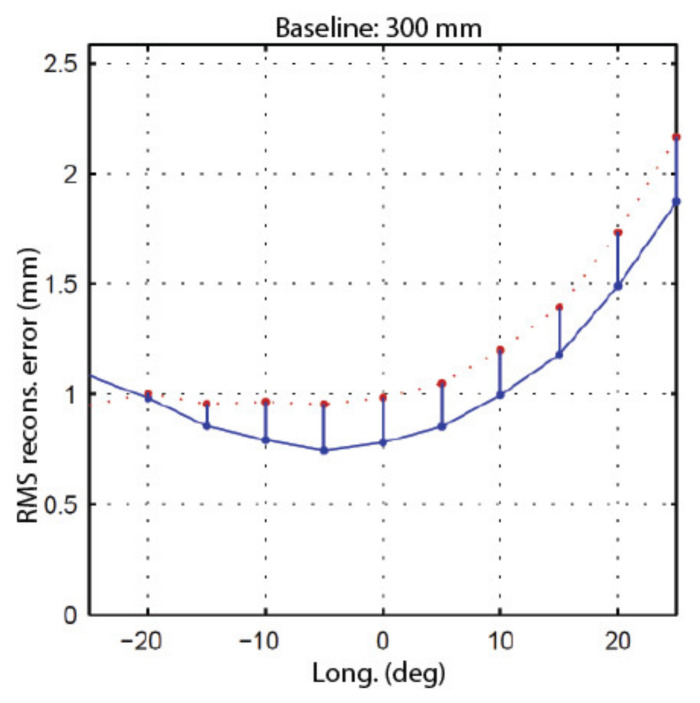
RMS reconstruction error of the 3D calibration target observed from the point of view located at 1.44 m from the ASP as a function of the direction of observation for a 300 mm baseline. Red dots: $E_{M_{R}C_{R}}=E_{M_{L}C_{L}}=I$, blue line $E_{M_{R}C_{R}}$, $E_{M_{L}C_{L}}$ calibrated with the proposed technique.

Figure 28 shows plots of the RMS error for the reconstruction of the 3D calibration target for 11 directions of observation. Solid lines show the reconstruction error obtained with transforms $E_{M_{R}C_{R}}$ and $E_{M_{L}C_{L}}$ calibrated with the speckle approach while dotted lines show the reconstruction error for $E_{M_{R}C_{R}}=E_{M_{L}C_{L}}=I$. Calibration of both $E_{MC}$ transforms achieves more accurate reconstructions than the one obtained with $E_{MC}=I$ for most of the points of view. This result is the first demonstration of the validity of calibration based on speckle metrology.

The second validation test compares the reconstruction results obtained with the ASP with the ones obtained by a Standard Stereo Pair (SSP). This experiment allows us to better isolate the role played by transforms $E_{M_{R}C_{R}}$ and $E_{M_{L}C_{L}}$ as well as the impact of the accuracy of the rotation encoders.

**Figure 29:**
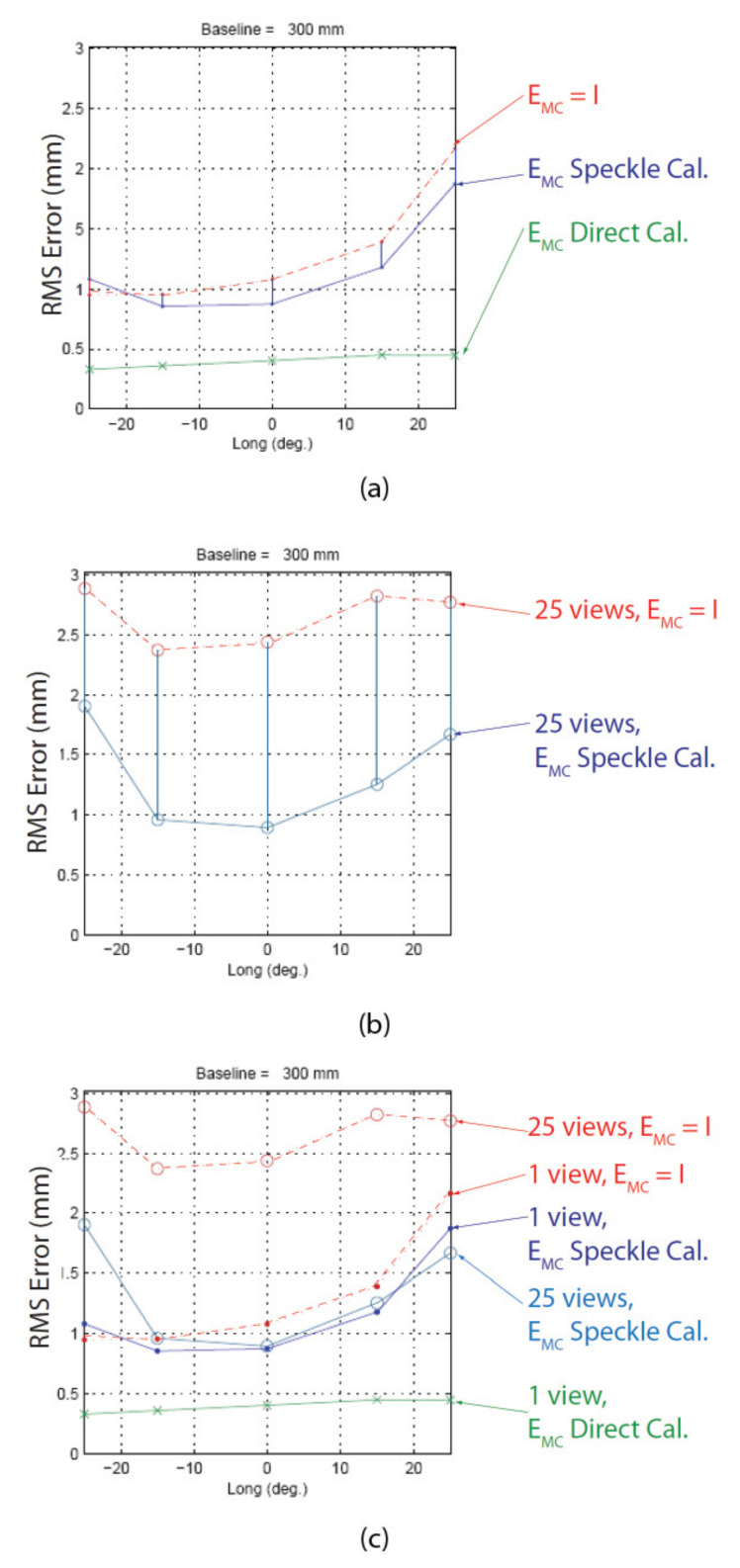
RMS reconstruction error of the discs on the calibration target for 5 different longitudinal orientations of the stereo pair. The red curve corresponds to $E_{MC}=I$, the blue to $E_{MC}$ calibrated with the speckle-based approach and the green to the ASP calibrated directly as a standard stereo pair (**a**). Reconstruction of the 3D target with 25 different views. The red curve corresponds to $E_{MC}=I$. The blue curve corresponds to $E_{MC}$ calibrated with the speckle-based approach (**b**). Superimposition of the curves in (**a**,**b**) on the same plot (**c**).

For this experiment, transform $E_{C_{R}C_{L}}$ was calibrated directly for five configurations of the ASP among the 11 directions of the previous experiment. To make sure that reconstruction results were compared under similar conditions, transform $E_{C_{R}C_{L}}$ was calibrated right after the acquisition of the images of the 3D calibration target were acquired. In other words, the cameras remained motionless between the moment when the images of the 3D target were acquired and the moment when the stereo pair was calibrated. The same location was then used for reconstructing the 3D target as a Standard Stereo Pair (*SSP*) and an *ASP*. Reconstruction results are shown in Figure 29a. A first observation is that, as expected, the reconstruction obtained with the SSP is more accurate than the one obtained with the ASP. Based on the way the experiment was conducted, the decrease in reconstruction accuracy for the ASP is caused by the calibration errors on $E_{M_{R}C_{R}}$ and $E_{M_{L}C_{L}}$ as well as the errors on the readings of the rotation encoders. Although the reconstruction experiments described above look convincing with respect to the ability of the speckle-based approach to calibrate the ASP with good reconstruction accuracy, as mentioned above, the registration process between ground truth data (measured with the CMM) and the reconstructed target was suspected of producing potentially biased results since the RMS error was used for computing registration and for assessing reconstruction accuracy afterwards.

To avoid the registration step, an additional experiment was conducted. Instead of measuring the position of the 25 discs of the calibration target from a single view of the ASP, the target was rather reconstructed from 25 different views, one for each disc of the calibration target. The orientation of each camera of the ASP was changed for each disc, so the image of the latter was located at the center of each image plane of the agile stereo pair. With this procedure, each disc is reconstructed from the ASP for a given configuration with a specific transform for each configuration. This transform uses all parameters listed in Table 4, including $E_{M_{L}C_{L}}$ and $E_{M_{R}C_{R}}$.

Now, if some configurations of the ASP yield an erroneous absolute value for the coordinates of a disc, this error will have a direct impact on the global reconstruction error while, when all 25 discs are reconstructed from a single view, an absolute measurement error affecting all measurements in a similar manner may remain unnoticed and may even be compensated for by registration before computing the RMS reconstruction error. Again, the reconstruction of the calibration target from 25 different views was repeated for five different configurations of the ASP, each with a 300 mm baseline. The results of this experiment are shown in Figure 29b, while Figure 29c superimposes the results of the experiments plotted in Figure 29a,b. This combination of the results shows that (*i*) the reconstruction accuracy of the approach using calibrated transforms $E_{M_{L}C_{L}}$ and $E_{M_{R}C_{R}}$ is practically unaffected by a continuous change in configuration of the ASP, which moves 25 times for achieving the reconstruction of the target and that (*ii*) the reconstruction error is important under the $E_{MC}=I$ hypothesis (where *I* is the identity matrix) and justifies the need for calibrating this transform.

### 5.4. Experiment 4: Calibration of a Two-Axis System

The calibration of a stereo pair, such as the ASP, made of two-axis mechanisms using speckle metrology was a major motivation for this work. This section is divided into two parts. The first part demonstrates that the sensitivity of the speckle-based calibration approach is such that it has allowed the detection of mechanical distortion of the ASP mechanisms during motion. A quantitative analysis of this distortion shows that the model of the ASP presented in Figure 4 would need improvements if it is to be used for better reconstruction. The second part describes the experiments conducted for calibrating a two-axis ASP system despite the mechanical distortions.

#### 5.4.1. Mechanical Distortion of the Two-Axis Mechanism

Alignment errors during the fabrication of the aluminum links composing the mechanisms are at the source of mechanical distortion when they are in operation. Since the design of the mechanisms is over-constrained, the parts with which they are fabricated must be perfectly assembled. As a matter of fact, in theory, if the links composing the mechanisms were perfectly rigid, even a small assembly error would prevent them from moving. In practice though, the compliance of the aluminum links allows motion even when the assembly is not perfect. The points where the links twist act as rotoid joints and transform a potentially blocked over-constrained mechanism into a free over-constrained one.

Based on the analysis that was made on the behavior of the mechanisms during complex simultaneous motion of their two degrees of freedom, bending and twisting of the links occur at many locations and the distribution of these locations changes dynamically with the configuration of the rotation axes. The end result of this non-ideal behavior is that the geometric model of the ASP presented in Figure 4 is not complete and that the development of an exact model accounting for the mechanical distortion would be challenging; however, as demonstrated in the following, this non-ideal behavior does not invalidate the speckle-based calibration approach, nor does it prevent reconstruction experiments with the ASP.

For exploring the distortion of the mechanism (the left and right eyes show similar behavior, and the experiment is described for the right eye only), two pairs of small retro-reflective circular markers were installed on the link actuated by the motor responsible for longitudinal displacements of the camera of the ASP since this link should not move during latitudinal motion of the mechanism.

**Figure 30:**
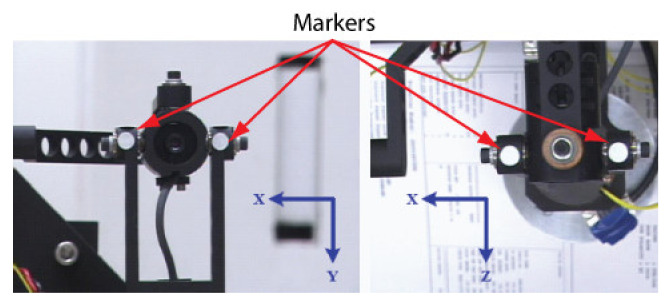
Markers installed on the longitudinal axis for observing mechanical distortion. Front markers (**left**). Top markers (**right**).

The first pair of markers was installed on top of the link, while the second pair was installed in front as shown in Figure 30 (both pairs of markers are located close to the camera so if they move, the camera is also bound to move). Each pair was observed by a stationary camera (not shown in Figure 30 and not to be mistaken with the one mounted in the mechanism) while executing a series of complex motions involving both rotation axes of the mechanism. This camera (resolution: 640 × 480) has been calibrated with Zhang’s algorithm prior to the experiment.

**Figure 31:**
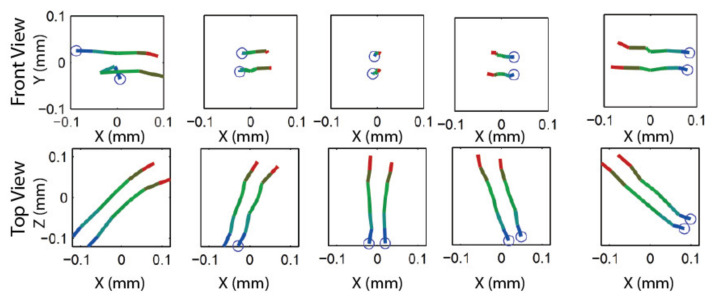
Effect of the mechanical distortion on the ASP.

In theory, each pair of markers should remain motionless during the movement of the mechanism; however, it is not the case at least for the translational motion caused by the mechanical distortion (rotational motion cannot be observed directly with the markers). The experiment consists of imposing a latitudinal motion to the mechanism (interval: −30° to 30°, step size: 10°) for a given longitudinal angular position and of measuring the position of the pairs of markers in the image of the front and top observation cameras. The results of this experiment for the right eye are shown in Figure 31. The top row shows the motion of the front markers, while the bottom row shows the motion of the top markers, each for a given longitudinal position. The color segments highlight the motion for each latitudinal angular position. To better observe the motion of both markers on the same plot, the curves have been centered at the null position of the mechanism (longitude = latitude = 0°). In addition, the curves have been displaced relative to each other, so they do not overlap. The circle corresponds to latitude −30°, which is the initial position of the mechanism. The displacement is along Y for the front markers and along Z for the top markers. The trajectories of the front and top makers demonstrate clearly that the right eye mechanism is submitted to significant deformation and that this deformation increases as the longitudinal angle is far from 0°. More precisely, the plots in Figure 31 show that the camera oscillates mostly from left to right (along the *X*-axis) and from front to back (along the *Z*-axis) during the latitudinal motion of the mechanism. It is interesting to compare the amplitude of the motion of the markers with the precision being achieved for the estimation of the translation components of $E_{MC}$.

Although the experiments allow the analysis of the motion along the *Z*-axis only, they show that the displacement of the markers along this axis can reach 0.1 mm and are close to ±0.04 mm in the angular interval used for the calibration of $E_{MC}$. Simulations of the mechanism show that the theoretical performance of the speckle-based calibration method for parameter $T_{MCZ}$ is 0.0012 mm. Consequently, the mechanical distortions are such that the positioning of the camera along the *Z*-axis is 30 times less accurate than what the calibration method can measure; however, the motion along the *X*-axis caused by the mechanical distortions is not bound to have any adverse effects on the speckle-based calibration technique since these distortions occur only when both axes are driven simultaneously. Now, as explained for the one-axis experiments presented in the previous sections and the two-axis calibration experiment presented in the next section, the calibration procedure implies that the sampling of angular positions is performed one axis at a time while the other axis remains stable at 0°. A conclusion on this may be that some effects of the mechanical distortions may remain undetected during calibration (since there is no displacement along X caused by distortion) but may have an adverse effect on 3D reconstruction since both mechanisms are actuated for measuring 3D data, and the distortions along X cause unmodeled (and uncalibrated) displacements of the cameras.

One might argue that displacements caused by mechanical distortions that remain unnoticed during calibration are a weakness of the speckle-based method; however, it is not the case since the calibration method has been designed to measure the parameters of the geometric model of the ASP shown in Figure 4. This geometric model does not include parameters for measuring mechanical distortion, so it is no surprise that the method does not provide any information on this aspect of the ASP. Should a more complete model including mechanical distortions be developed, it is likely that the speckle-based calibration method could be adapted for estimating the additional parameters describing the motion of the camera caused by distortions (for instance, by using both axes simultaneously during calibration) since rotation and translation can be decoupled by the approach; however, the development of a geometric model including mechanical distortion is not an easy task and has not yet been investigated further on the current implementation of the ASP.

#### 5.4.2. Result of the Calibration of a Two-Axis ASP System

The experiment described in this section aims at demonstrating the calibration of a two-axis ASP. The demonstration is also made that transform $E_{MC}$ can be estimated with good repeatability for the ASP in such a configuration. Since the calibration of a two-axis ASP is an extension of the one-axis case, several fundamental aspects of the speckle-based calibration method have already been validated in Experiment 2. The only aspect specific to the two-axis system that remains to be validated is the simultaneous non-linear optimization leading to the estimation of the geometric model parameters. For a two-axis system, the setup shown in Figure 16a is used, and the first three steps are similar to those used for the one-axis system: (*i*) adjustment of the focus of the camera at infinity, (*ii*) calibration of the intrinsic parameters using Zhang’s approach [25] and (*iii*) positioning of the APSS in front of the camera.

At this point of the procedure, three steps differ from the one-axis case. Firstly, the sampling of angular positions is performed on two axes. Secondly, the sampling of the motion of the camera (STEP 2) is performed from a single point of view for each axis. That is: the additional points of view shown in Figure 24b for the one-axis method are not used. Finally, an extra parameter, $\theta_{Skew}$, describing the angle between the two axes of the mechanisms is included in the calibration (and non-linear optimization process) since it is very unlikely that they are perpendicular.

STEP 1

The adjustment of the focus of the camera as well as the positioning of the APSS in front of the mechanism do not imply any motion of the axes and the results are the same as those obtained for the one-axis system.

STEP 2

The sampling of the angular displacements of the ASP has been performed on the interval between −8° to +8° for the longitudinal axis and for the interval between −5.3° to +5.3° for the latitudinal axis. The sampling step was 0.2° for both axes. The sampling interval for the latitudinal axis is smaller due to the resolution of the camera in this direction (480 pixels vs. 640 pixels for the longitudinal axis). As in Experiment 2 for the one-axis case, secondary sweeps and tracking of multiple speckle points were implemented.

STEPs 3 and 4

**Figure 32:**
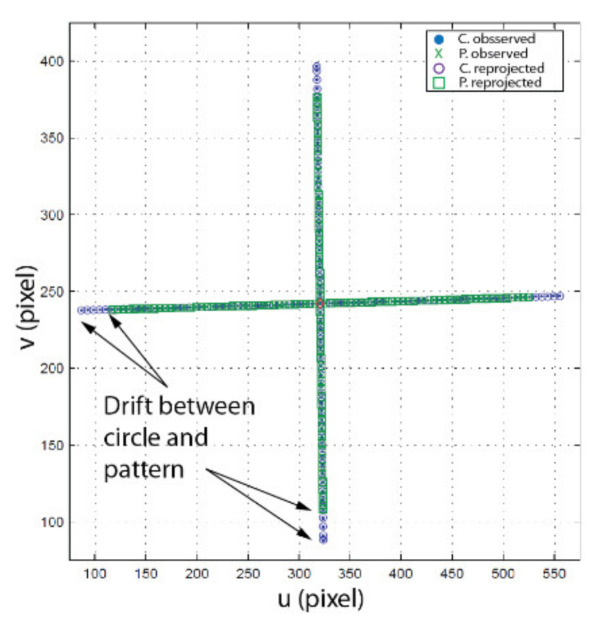
Result for the calibration of a two-axis mechanism showing the observed and reprojected position of the circle and pattern. The drift between the circle and pattern is visible on both axes.

The information collected at STEPs 1 and 2 is then used at STEPs 3 and 4 for estimating the rotation and translation components of transform $E_{MC}$ according to the non-linear optimization procedure already discussed in Figure 19. The results for the reprojection of the speckle and circle (central sweep only) for the right eye are shown in Figure 32 for the longitudinal and latitudinal axes (the rotation of the camera around its optical axis has also been corrected).

**Figure 33:**
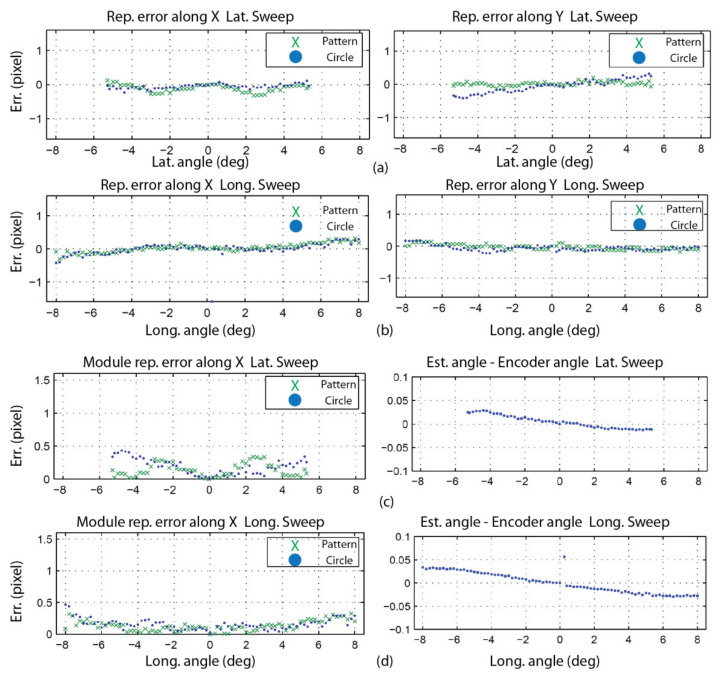
Results of the calibration of a two-axis system for the right eye showing the reprojection error for the principal sweep along the latitudinal axis (**a**) and the longitudinal axis (**b**). One can check that the error is smaller than 0.5 *pixels* for each axis. Module of the reprojection error and difference between the estimated angle and the angle read on the encoders for the latitudinal axis (**c**) and the longitudinal axis (**d**).

Figure 33 shows the details of the reprojection errors of Figure 32. The first observation is that the reprojection error is small (less than 0.5 pixels) for both axes. This is proof that the geometrical model of the ASP explains the observations well despite mechanical distortions. The validity of the non-linear optimization procedure for calibrating *E_MC_* for a two-axis system is also implicitly validated by these results.

Several other observations can be made from these results. For instance, the reprojection error of the circle along the *Y*-axis during latitudinal rotations shows a systematic linear bias. It is suspected that the mechanical distortions are responsible for this bias and could be linked to the backward–forward motion of the mechanism observed in Figure 31. As a matter of fact, the discrepancy between the anticipated drift of the position of the circle and the measured drift could be explained by a translation that is not accounted for by the geometrical model of Figure 4, which is exactly the case for rotations around the latitudinal axis. One might argue that errors in the measurement of angles could also be at the origin of the reprojection errors of the circle. Indeed, the plot of Figure 33d shows a linear bias in the measurement of the angles; however, this bias cannot be the cause of the reprojection error of the circle because it is opposite to what it should be to explain the plot of the reprojection error. It must be noted that the plots in Figure 33d show that the magnitude of angular measurements estimated by the speckle-based approach is overestimated when compared to encoder readings. Such an overestimation of the angles should result in positive reprojection errors for negative orientations and negative reconstruction errors for positive orientations, a behavior that is contradicted by experiments. The mechanical distortions are rather suspected of being responsible for these results.

The last point that needs to be explained is precisely the linear bias on the error in the measurement of angles by the speckle-based approach when compared to encoder readings. It is hypothesized that if the angular displacements were poorly estimated by the speckle-based approach, this estimation error would be observed for the reprojection error of the circle. Now, the reprojection error of the circle is almost zero for the longitudinal axis. It has been mentioned above that this reprojection error associated with the latitudinal axis cannot be explained by errors in angular measurements. It is thus suspected that such results also support the evidence of mechanical distortions occurring during the calibration experiment.

The careful reader may have noticed the outlier measurement for the longitudinal sweep on the plot of Figure 33d. The source of this result was analyzed and can be explained by a measurement error caused by a loss of synchronization between image acquisition and the instruction sent to the mechanism for imposing an angular displacement. This outlier event is thus not linked to the speckle-based calibration procedure. The calibration experiment on a two-axis system was repeated four times for both the right and left eyes of the ASP. Table 8 shows the results of these experiments for the right eye. Although such a small number of experiments is not statistically significant, it shows that the speckle-based approach produces results that are repeatable with an acceptable level of precision; however, because of the mechanical distortions, the accuracy of the method for 3D stereo reconstruction cannot be assessed for the current prototype of the ASP. Despite the small values of the reprojection errors reported above, the lack of confidence in the accuracy of the estimated $E_{MC}$ transforms for a two-axis system would lead to erroneous conclusions on the reconstruction error of the 3D target, contrarily to what was conducted for the one-axis system and, consequently, this experiment is not presented here.

## 6. Conclusions and Future Work

In this paper, an approach for the geometric calibration of cameras in active stereo pairs using laser speckle is presented. The calibration approach, which was tested on a device called the Agile Stereo Pair (ASP), shows that it achieves better accuracy than standard calibration methods. Although the speckle-based calibration method is tested on the ASP, it extends nicely to other systems exploiting tilt/pan mechanisms. The speckle-based calibration procedure decouples translation from rotation in the optimization process and allows to achieve better 3D reconstruction

In future work, the speckle-based calibration approach could be exploited for other camera calibration tasks. Firstly, the approach could be used for the calibration of active stereo systems different from the ASP. Indeed, since the approach allows to decouple rotation from translation, it is general and can be used for any mechanism for which small rotations and translations need to be estimated accurately. Secondly, speckle-based calibration could be used with Zhang’s approach by replacing the standard checkerboard target with an array of Almost Punctual Speckle Sources.

## Figures and Tables

**Table 1:** Details on the components of the ASP.

Component	Description
Cameras	Toshiba SM-43 NTSC, 7 mm ϕ, weight = 9 g, 1/4′′ CCD, Field of view = 30°
Translation stages	Newport MTM250OCC1, res.: 1 μm, Baseline variation range (Λ in cm): 5≤Λ≤50
Rotation encoders	MicroE M1500 (327,680 steps/rev)
Driving motors	24 v DC
Real-time control of the motors	QNX-OS

**Table 2:** Dynamic performance of the ASP.

DOF	Range	Resolution	Angular/Linear Speed	Angular Acceleration
pan	±40°	0.0011°	1950°/s	78,000°/s^2^
tilt	±40°	0.0011°	1350°/s	40,000°/s^2^
baseline	50 cm	1 μm	8 cm/s	

**Table 3:** Definition of parameters of the geometric model of the cameras.

Parameter	Definition	Type ^a^
*Q*	rotation matrix describing the rotation between frame *O_C_* and frame *O_W_*	E
*t*	translation vector describing the translation between frame *O_C_* and frame *O_W_*	E
*f*	focal length of the pinhole model	I
$s_{x}$ $,s_{y}$	scale factors along the x and y axes	I
*θ*	angle between the *x* and *y* axes of the image plane	I
$u_{0},v_{0}$	coordinates of the center of the image plane	I
*α*	$s_{x}f$	I
*β*	$s_{y}f/\sin\left(\theta \right)$	I
*γ*	$-s_{y}f/\tan\left(\theta \right)$	I

^a^ E: extrinsic, I: intrinsic.

**Table 4:** List of parameters of the ASP to be calibrated.

Category	No. of Parameters	No. of Instances	Total
Intrinsic parameters of a camera (Figure 3)	7	2	14
Transform $E_{MiCi}$ (*i* = *r*,*l*) (Figure 4)	6	2	12
Translation axis $L_{i}$ (Figure 4)	3	2	6
Transform $E_{RrRl}$ (Figure 4)	6	1	6
**Total**			**38**

**Table 5:** Implementation of the APSS.

APSS Parameter	Value
Laser	30 mW @ 635 nm
Thickness of diffusing material	10 pages of Vellum paper
Pinhole diameter	100 μm
Pinhole thickness	26 μm
Thickness—Frosted plexiglass mask	6 mm

**Table 6:** Experimental conditions for Experiment 1.

Parameter	Value
angular interval for rotations	−6.5° to + 6.5°
angular step size	0.2°
point of the speckle pattern used for computing the direction vector for tracking	speckle point at the center of the circle when the camera is at 0°
diameter of the image of the circle	200 pixels
correlation window for finding the speckle pattern	41 × 41 pixels

**Table 7:** Results for three independent calibration experiments (left eye).

Cal.	Parameter				Rep. Err. (RMS)	
	$T_{MCx}\;(\mathrm{mm})$	$T_{MCz}\;(\mathrm{mm})$	$R_{MCx}\;(\deg.)$	$R_{MCz}\;(\deg.)$	Pattern (Pixel)	Circle (Pixel)
1	0.5104	−3.1823	0.7211	1.8537	0.23	0.24
2	0.5357	−3.1802	0.7624	1.8585	0.24	0.26
3	0.4335	−3.1815	0.7931	1.8579	0.24	0.21
$\bar{\mu }$	0.4932	−3.1813	0.7589	1.8567	0.24	0.24
σ	0.0434	0.0009	0.0295	0.0021		

**Table 8:** Calibration results for 4 different experiments on a two-axis mechanism (right eye).

Cal	Parameters							Rep. Err.	
	$T_{CM}(\mathrm{mm})$			$T_{CM}(\deg)$			(deg)	(Pixel)	
	X	Y	Z	X	Y	Z	$\theta_{Skew}$	Pat.	Cir.
1	−0.1269	0.0911	−3.5448	−0.9676	0.3396	1.1288	−0.0465	0.17	0.23
2	−0.1332	−0.2707	−3.5350	−1.0285	0.1128	1.1370	−0.0459	0.18	0.17
3	−0.0559	0.0821	−3.5470	−1.0336	0.3863	1,1333	−0.0420	0.17	0.17
4	0.1283	−0.0335	−3.5427	−0.9462	0.3984	1.1383	−0.0344	0.18	0.19
*μ*	*−0.0469*	*−0.0327*	*−3.5424*	*−0.9940*	*0.3093*	*1.1344*	*0.0422*	*0.17*	*0.19*
*σ*	*0.1056*	*0.1459*	*0.0045*	*0.0379*	*0.1155*	*0.0037*	*0.0048*		

**Figure A1:**
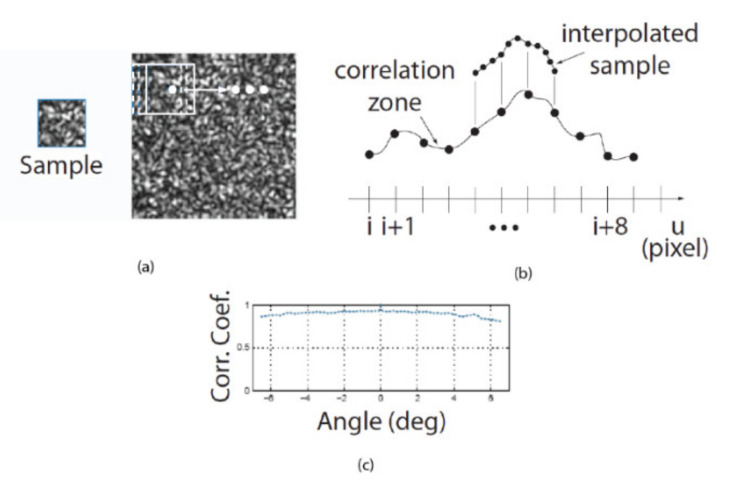
Sample of the speckle pattern and sweeping of the sample over the pattern (**a**). Sub-pixel interpolation of the position of the sample for which the normalized correlation coefficient is maximum (**b**). Typical plot of the evolution of the correlation coefficient as a function of the angle for one axis of one eye of the ASP (**c**).

**Figure A2:**
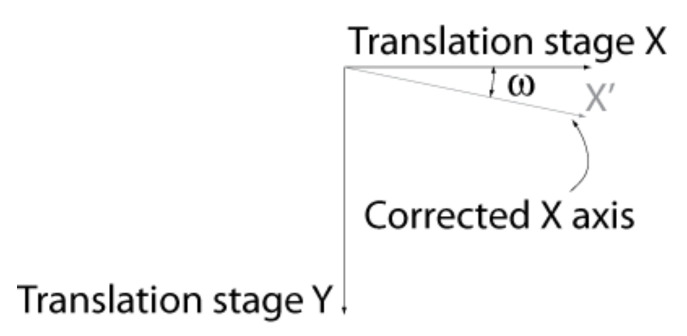
Non-orthogonality between the translation stages expressed by parameter *ω*.

## Appendix A

Computation of the Center of Gravity of the Circle

The position of the center of gravity of the circle is computed using a three-step procedure. The first step consists of finding pixels located on the contour of the circle with the Canny edge detector [47]. Pixels returned by the Canny operator correspond to the maximum amplitude of the gradient. The position of these contour pixels is refined by finding, in a second step, their sub-pixel position along the direction of the image gradient. The third step finally consists of fitting a circle on the refined contour points. The parameters of the fitted circle provide the location of its center.

## Appendix B

Detection of the Speckle Pattern

An important step in the calibration process consists of detecting the motion of the speckle pattern caused by the rotation of the camera. The approach for detecting this motion is a three-step procedure. The first step consists of choosing a sample of the speckle pattern. This sample is a square region circumscribing the center of the circle in the reference position as shown in Figure 18. In a second step, the sample is then swept one pixel at a time for each line of the circle at the current angular position of the camera (Figure A1a) and the normalized correlation coefficient (Equation (A1)) is computed for each position of the sample.

(A1) $$\gamma \left(u,v\right)=\frac{C}{D}$$

with

(A2) $$C=\overset{W}{\underset{m=-W}{\sum }}\overset{W}{\underset{n=-W}{\sum }}\left[I\left(u+m,v+n\right)-{\langle I\rangle }_{\left(u,v\right)}\right]\left[E\left(m,n\right)-\langle E\rangle \right]$$

and

(A3) D=∑m=−WW∑n=−WWIu+m,v+n−〈I〉u,v2∑m=−WW∑n=−WWEm,n−〈E〉212

In Equation (A2), *W* is the size of the sample, *I* is the illuminance of the image, ${\langle I\rangle }_{\left(u,v\right)}$ is the average illuminance in the neighborhood *W × W* of (*u*,*v*), *E* is the illuminance at pixels in the sample and 〈E〉 is the average illuminance of the sample. The normalized correlation coefficient is robust to illuminance variation from one image to the other. The pixel $(u_{max},v_{max}$) for which $\gamma \left(u,v\right)$ is maximum is considered as the location of the sample in the speckle pattern. The precision of the estimate of the location of the maximum is up to a pixel only. This is not enough for achieving accurate calibration. A sub-pixel procedure is thus implemented as a third step. The sample is interpolated to 1/10th of a pixel using bicubic splines and the sub-pixel sample is correlated with the image around $(u_{max},v_{max}$). At each position of the interpolated sample, only the pixels of the pattern corresponding to the native resolution are considered for computing the correlation coefficient. This procedure is illustrated in 1D (and for a 1/3rd interpolation) in Figure A1. In the current implementation of the calibration procedure, W=41. This value is a tradeoff between accurate detection of the position of the sample over the pattern and the size of the speckle pattern in the circle. Choosing a larger value for *W* would reduce the effect of noise at the cost of increasing the region of the circle for which the sample exceeds the border of the circle, which affects the computation of the correlation coefficient.

## Appendix C

Correction of the Non-Orthogonality between the Linear Translation Stages in the Estimation of the position of the APSS in the Reference Frame of the Camera

It is possible to account for the non-orthogonality between the translation stages in the experiment for estimating the position of the APSS in the reference frame of the camera at STEP 1 of the calibration process (Section 4.3). To achieve this, a transform $E_{Skew}$ expressed as:

(A4) $$E_{Skew}=\left[\begin{array}{cccc} \cos\left(\omega \right) & 0 & 0 & 0\\ \sin\left(\omega \right) & 1 & 0 & 0\\ 0 & 0 & 1 & 0\\ 0 & 0 & 0 & 1 \end{array}\right]$$

is used, where *ω* is the angle between the linear stages (see Figure A2).

With this new parameter to calibrate, Equation (15) becomes:

(A5) $$s\left[\begin{array}{c} u_{i}\\ v_{i}\\ 1 \end{array}\right]=KE_{CW}E_{Skew}\left[\begin{array}{c} X_{Wi}\\ Y_{Wi}\\ Z_{Wi}\\ 1 \end{array}\right]$$

The estimation of ω requires a modification of the procedure for estimating the pose of a camera in the reference frame of the calibration target (see Section 4.2). In this case, the set of parameters to be optimized in Equation (25) becomes $\Omega =\left\{\theta ,\psi ,\gamma ,t_{x},t_{y},t_{z},k_{1},k_{2},\omega \right\}$.
